# An RNA thermogenic therapy to preserve lean mass and enhance metabolic health during GLP-1 weight loss

**DOI:** 10.1073/pnas.2618845123

**Published:** 2026-09-25

**Authors:** Anneke K. Thorne, Nathan J. Waldeck, Jesse P. Rose, Marisa J. Stachowski, Hannah Guak, Alexander R. Keeble, Britta Kuusik, Ella Simon, Biliana Marcheva, Joseph V. Mastroni, Pei Zhu, Ling Wa Chong, Yumiko Kobayashi, Nicholas J. Conley, Andrew B. Cohen, Yasuhiro Omura, Chiaki Omura, Weimin Song, Michael Wellems, Kathryn M. Ramsey, Grant D. Barish, Lisa R. Beutler, Rana K. Gupta, Clara B. Peek, Elizabeth M. McNally, Chelsea Hepler, Mark Perelis, Joseph Bass

**Affiliations:** ^a^Department of Medicine, Division of Endocrinology, Metabolism, and Molecular Medicine, Feinberg School of Medicine, Northwestern University, Chicago, IL 60611; ^b^Center for Genetic Medicine, Feinberg School of Medicine, Northwestern University, Chicago, IL 60611; ^c^Department of Biochemistry and Molecular Genetics, Feinberg School of Medicine, Northwestern University, Chicago, IL 60611; ^d^Ionis Pharmaceuticals, Carlsbad, CA 92010; ^e^Phenome Technologies Inc., Lincolnshire, IL 60091; ^f^ Division of Endocrinology, Department of Medicine, Duke Molecular Physiology Institute, Duke University, Durham, NC 27701; ^g^ Department of Molecular and Integrative Physiology, University of Michigan, Ann Arbor, MI 48109

**Keywords:** adipose thermogenesis, ZFP423, antisense oligonucleotide, GLP-1 receptor agonist, obesity

## Abstract

Current obesity therapies reduce weight but often fail to preserve lean mass or sustain metabolic benefit. Glucagon-like peptide-1 receptor agonists (GLP-1RA) improve obesity-associated metabolic disease, but weight loss is often accompanied by reduced energy expenditure, loss of lean mass, and rapid fat-predominant regain after treatment discontinuation. We hypothesized that pharmacologic activation of adipose thermogenesis could specifically target fat loss, complement appetite suppression by increasing energy expenditure, and improve the metabolic quality of weight loss. Guided by our finding that diet-induced thermogenesis is mediated by a zinc finger protein 423 (ZFP423)-controlled pathway, we developed adipose-directed antisense oligonucleotides (ASOs) targeting ZFP423, a transcriptional repressor of brown and beige adipocyte identity. In lean and diet-induced obese mice, weekly *Zfp423* ASO treatment induced white adipose beiging, increased body temperature and oxygen consumption, and improved glucose homeostasis, insulin sensitivity, lipid metabolism, mitochondrial respiration, and hepatic steatosis. When combined with the GLP1-RA semaglutide, *Zfp423* ASO produced greater weight and fat loss than either monotherapy while preserving lean mass and improving metabolic outcomes. These findings identify *Zfp423* as an RNA therapeutic target and support adipose thermogenesis as a complementary strategy for metabolically healthier obesity treatment.

## Significance

Current antiobesity therapies reduce body weight but often induce loss of lean mass and fail to achieve durable metabolic improvements after treatment ends. We show that an adipose-targeted RNA therapeutic targeting ZFP423, a transcriptional repressor of brown and beige fat identity, enhances thermogenesis and energy expenditure to complement glucagon-like peptide-1 receptor agonist (GLP-1RA) treatment. Suppression of ZFP423 promotes white adipose beiging and improves systemic metabolism in mice, and when combined with GLP-1RA produces greater fat loss than either treatment alone while preserving lean mass. These results identify *Zfp423* as an RNA therapeutic target and leverage adipose thermogenesis as a clinical adjuvant to GLP-1RA for obesity pharmacotherapy.

Obesity is a chronic disease of energy imbalance in which increased energy intake and dysregulated energy expenditure promote the defense of an elevated body weight. The discovery of leptin as an adipostatic hormone established a mechanistic framework for how weight loss is strongly opposed biologically, with diminished leptin signaling after weight loss triggering increased hunger, reduced energy expenditure, and adaptive neuroendocrine changes (1). Obesity, therefore, is not simply a consequence of excess caloric intake, but reflects biological adaptations such as suppression of thermogenesis to conserve energy stores (2). Emerging evidence suggests that impaired thermogenic capacity and suppression of energy dissipation contribute to metabolic inflexibility and sustained adiposity (3, 4). The metabolic consequences of obesity are closely linked to expansion of proinflammatory visceral adipose tissue relative to lean mass, contributing to insulin resistance and comorbidities, including hepatic steatosis or metabolic dysfunction-associated steatotic liver disease (MASLD), cardiometabolic disease, stroke, and cancer (5–9). Weight regain after weight loss often worsens metabolic risk because regain is often characterized by adipose tissue expansion without recovery of lean mass, promoting glucose and lipid intolerance and increasing long term disease risk (10, 11).

Incretin-based therapies have revolutionized obesity treatment by bypassing physiologic constraints on appetite regulation, producing substantial weight loss and improving metabolic disease (12–14). However, they are limited by reductions in energy expenditure, loss of lean mass, and weight regain upon treatment cessation, as they do not address the underlying biological systems that maintain elevated body weight (15–17). Thus, despite their transformative clinical success, achieving metabolically durable weight loss remains a central challenge.

We recently discovered that the timing of nutrient availability profoundly influences energy balance through circadian control of adipose thermogenesis (18). Time-restricted feeding, which synchronizes eating patterns to endogenous circadian rhythms, enhances energy expenditure and metabolic health independent of energy intake or lean mass, partly by enhancing adipose thermogenesis during the active period (nighttime for mice) (18, 19). Human studies similarly show altered diurnal energy expenditure with sleep loss, circadian misalignment, and obesity (20).

A key finding from our study was that knocking out the transcription factor zinc finger protein 423 (ZFP423) prevented the negative effects of feeding mice a high fat diet during their inactive period (18). Mice with reduced ZFP423 activity were healthy regardless of when they consumed calories. ZFP423 is a transcriptional repressor of brown and beige adipogenesis (21–23) and regulates activation of early B cell factor 2 (EBF2) at thermogenic gene enhancers (24–26). Genetic studies have shown that suppression of ZFP423 in white adipose tissue restores thermogenic capacity and protects against excessive weight gain and metabolic dysfunction during high fat feeding (21–23, 27–29). In humans, metabolic health is associated with the abundance and activity of thermogenic adipose tissue, supporting enhanced energy expenditure as a potential therapeutic strategy to improve beta-oxidation and systemic metabolism (30–33). Building on these findings, we developed an adipose-targeted antisense oligonucleotide (ASO) strategy to suppress ZFP423 in adult animals to mimic the prothermogenic effects of time-restricted feeding.

Here we tested whether adipose-directed suppression of ZFP423 can overcome key limitations of current obesity therapies. We asked whether pharmacologic activation of adipose thermogenesis could enhance energy expenditure and metabolic flexibility, reduce hepatic steatosis, preserve lean mass during weight loss, and augment GLP-1RA treatment in vivo while offsetting metabolic liabilities. Together, our results identify ZFP423 as a therapeutic target for the selective reduction in adiposity and preservation of lean mass to combat obesity.

## Results

### Screening Identifies Well-Tolerated Adipose-Targeted *Zfp423* ASOs With Robust Target Knockdown.

**Fig. 1.**
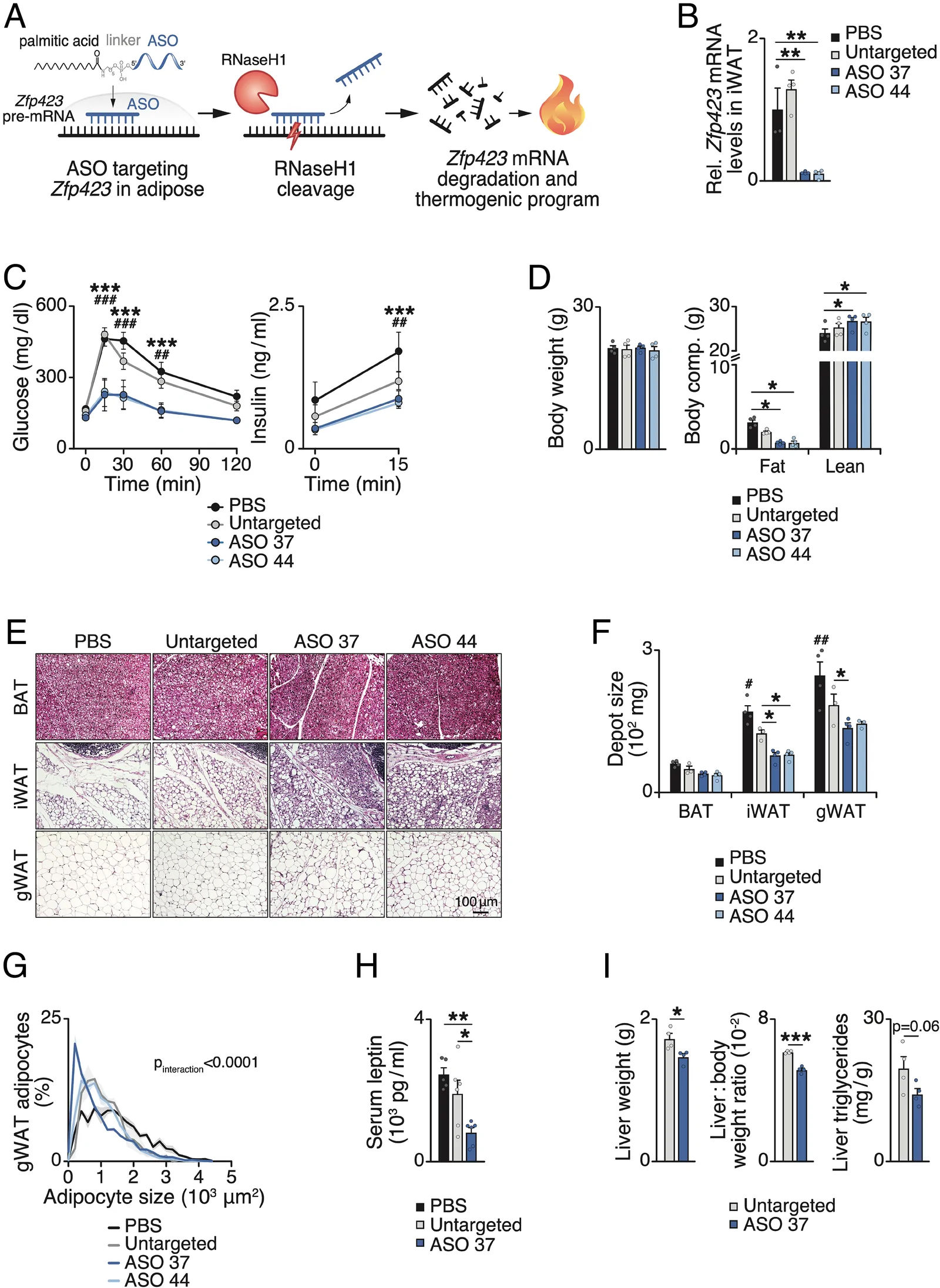
Adipose-targeted suppression of *Zfp423* enhances thermogenesis and metabolic health. (*A*) Schematic of adipose-targeted delivery of palmitic acid-conjugated antisense oligonucleotide (ASO) targeting *Zfp423* pre-mRNA to reduce *Zfp423* expression and promote thermogenesis. (*B*) Relative *Zfp423* mRNA expression in iWAT after 10 wk of weekly injections of PBS, untargeted ASO, ASO37, or ASO44 (50 mg/kg/dose) in mice fed a chow diet (n = 3 to 4). (*C*) Glucose tolerance test (GTT) and plasma insulin at ZT14 after 4 wk of ASO treatment (n = 4). * and # indicate significance for ASO37 and ASO44, respectively, relative to PBS. (*D*) Body weight and composition (n = 4) and (*E*) representative hematoxylin and eosin (H&E) staining of BAT, iWAT, and gWAT after 10 wk of treatment. (*F*) Adipose depot weights; #*P* < 0.05 and ##*P* < 0.01 relative to all other treatment conditions in iWAT and gWAT (n = 4). (*G*) Distribution of gWAT adipocyte size (n = 4). (*H*) Serum leptin at necropsy (n = 5 to 6). (*I*) Liver weight, liver:body weight ratio, and liver triglycerides (n = 4). Data are mean ± SEM. Statistics: one-way ANOVA with Tukey correction for one categorical independent variable, two-way ANOVA with Dunnett correction for two categorical independent variables, or Student’s *t* test for two-group comparisons. *P < 0.05, **P < 0.01, *** or ###*P* < 0.001.

To pharmacologically target ZFP423 in adipose tissue, we developed adipose-directed *Zfp423*-targeting antisense oligonucleotides (ASOs). To enhance adipose tissue delivery, ASOs were conjugated to a palmitic acid linker (Fig. 1*A*) (34, 35). Twelve *Zfp423*-targeting ASOs spanning the *Zfp423* transcript were generated and screened in vivo for knockdown efficacy and tolerability (*SI Appendix*, Fig. S1 *A* and *B*). Mice received three subcutaneous injections over ten days at 50 mg/kg per dose, and tissues and serum were collected after the final dose to assess target knockdown efficiency and systemic safety (*SI Appendix*, Fig. S1*C*). *Zfp423* expression in epididymal adipose tissue was quantified by qPCR. Among the candidates, ASO36, ASO37, ASO38, ASO44, and ASO47 produced greatest reductions in adipose *Zfp423* expression (*SI Appendix*, Fig. S1*D*). Systemic tolerability was evaluated by monitoring body weight and serum chemistry. Body weight remained stable across the treatment groups, suggesting no overt systemic toxicity (*SI Appendix*, Fig. S1*E*). Liver-associated markers, including aspartate and alanine amino transferases (AST and ALT, respectively), bilirubin, and albumin, showed no broad evidence of hepatic dysfunction across most ASO-treated groups with the exception of ASO38, ASO42, and ASO43, which had elevated AST and ALT (*SI Appendix*, Fig. S1*F*). Kidney-associated markers, including creatinine and blood urea nitrogen, were also assessed, and all candidates except ASO38 showed no evidence of renal dysfunction (*SI Appendix*, Fig. S1*F*). Based on both knockdown efficacy and tolerability, ASO37 and ASO44 were selected for further study.

### Adipose-Targeted Suppression of *Zfp423* Enhances Thermogenesis and Metabolic Health.

To evaluate the metabolic effects of sustained ZFP423 suppression, chow-fed mice were treated weekly for 10 wk with PBS, an “untargeted” ASO control which does not bind to any known RNA, ASO37, or ASO44 (Fig. 1 *A* and *B*) (34, 35). *Zfp423* knockdown remained effective after longer term dosing (Fig. 1*B*). Within 4 wk, both ASO37- and 44-treated mice exhibited improved glucose tolerance and insulin sensitivity compared to controls (Fig. 1*C*) even while total body weight remained unchanged (Fig. 1*D*). Body composition analyses revealed reduced fat mass with a concomitant increase in lean mass (Fig. 1*D*). Histological analysis revealed remodeling of adipose tissue depots. Inguinal white adipose depots (iWAT) from ASO-treated mice showed significant beiging, characterized by increased multilocular adipocytes (Fig. 1*E*) (36–38). In contrast, gonadal adipose tissue (gWAT) did not show significant beiging but exhibited reduced depot mass and adipocyte size (Fig. 1 *E*–*G*), consistent with a metabolically healthier adipose phenotype (39). There is a small loss of adiposity with the untargeted condition; however, beiging and metabolic improvement are unique to ZFP423 treatment (Fig. 1 *C*–*G*) (40). Serum leptin was significantly reduced relative to both controls (Fig. 1*H*). Liver weight and liver-to-body weight ratio were also significantly reduced, with a trend toward reduced hepatic triglyceride content under chow-fed conditions (*P* = 0.06) (Fig. 1*I*).

### Adipose-Targeted *Zfp423* ASOs Increase Thermogenic Energy Expenditure.

**Fig. 2.**
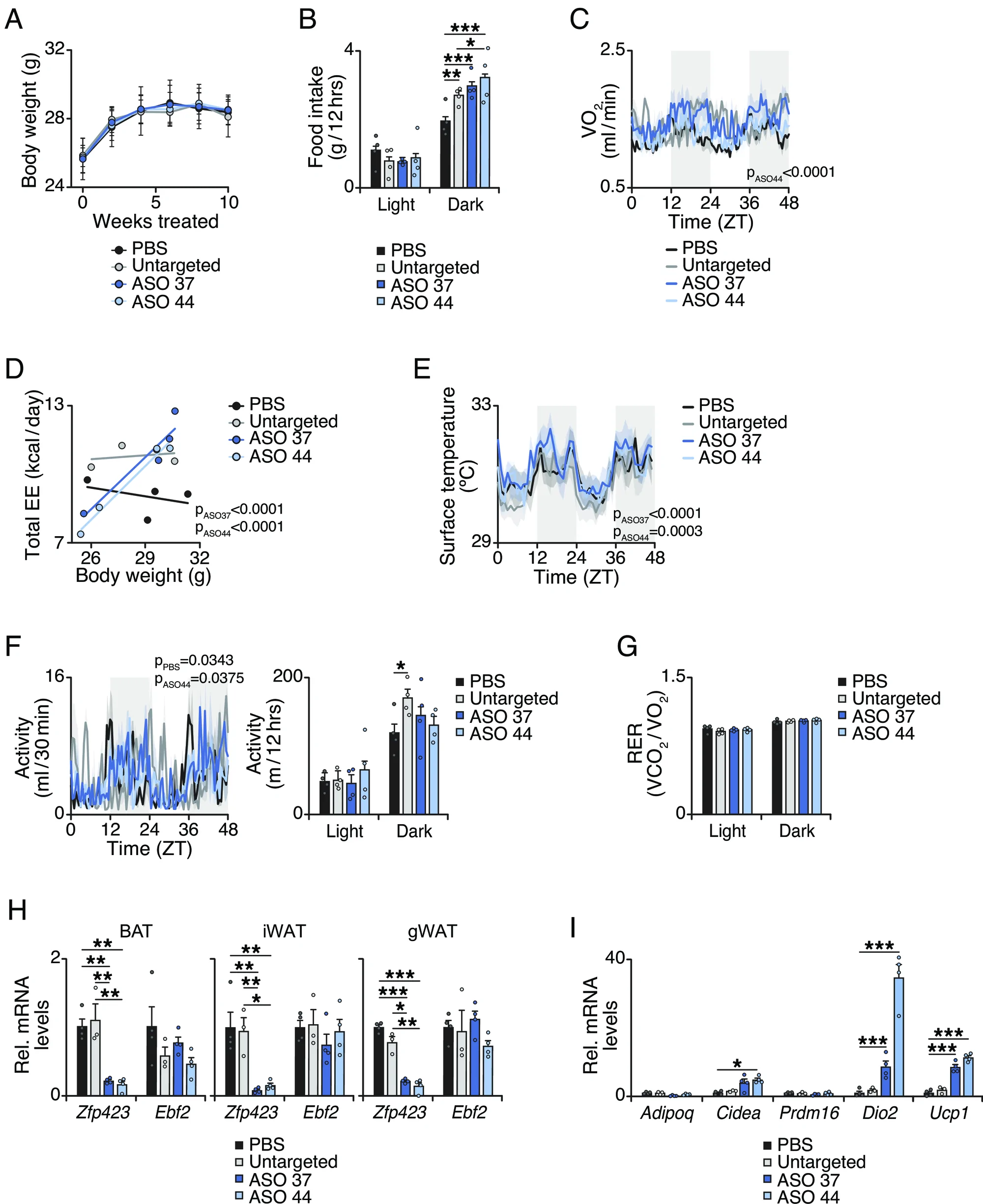
Adipose-targeted *Zfp423* ASOs increase thermogenic energy expenditure. (*A*) Body weight throughout the 10-wk ASO dosing protocol on chow diet (n = 4). (*B*) Light- and dark-period food intake and (*C*) oxygen consumption traces after 8 to 9 wk (n = 4). Pairwise *P* values on plots are relative to the untargeted treatment throughout. P relative to PBS: P_untargeted_ < 0.0001, P_ASO37_ < 0.0001, P_ASO44_ = 0.01 (*D*) 24-h energy expenditure versus body weight analyzed by Gaussian generalized linear model including treatment, body mass, and their interaction; slopes were estimated with emtrends and compared pairwise with Sidak-adjusted *P*-values. (n = 4). P relative to PBS: p_ASO37_ < 0.0001, p_ASO44_ < 0.0001. (*E*) Surface body temperature by thermal imaging after 6 to 7 wk (n = 4). P relative to PBS: P_ASO37_ < 0.01. (*F*) Activity traces and activity during the light and dark phases (P relative to PBS: P_untargeted_ < 0.05), and (*G*) respiratory exchange ratio (VCO_2_/VO_2_) during the light and dark phases during weeks 8 to 9. (*H*) Relative *Zfp423* and *Ebf2* mRNA levels across adipose depots (n = 3 to 4). (*I*) Relative mRNA levels of adipose and beige markers in iWAT (n = 3 to 4). Data are mean ± SEM or estimated marginal mean ± SEM for calorimetry plots. Statistics: one- or two-way ANOVA with Tukey correction. *P < 0.05, **P < 0.01, ***P < 0.001.

Body weights did not diverge among mice treated with PBS, an untargeted ASO, ASO37 or ASO44 during 10 wk of chow-diet dosing, despite increased food intake during the active phase (dark period for mice) relative to PBS (Fig. 2 *A* and *B*). These findings suggested that enhanced energy expenditure, rather than reduced caloric intake, contributed to the improved metabolic phenotypes. To test this hypothesis, mice were monitored in calorimetry metabolic phenotyping cages during weeks 8 to 9 of treatment. Oxygen consumption was significantly increased in *Zfp423* ASO-treated mice, particularly during the active period (Fig. 2*C*). Analysis of energy expenditure by Gaussian generalized linear model (GLM), with body mass as a covariate, revealed an increased slope of energy expenditure relative to body mass in ASO-treated mice, indicating enhanced thermogenesis independent of body size, consistent with increased surface body temperature (Fig. 2 *D* and *E*). Locomotor activity in ASO-treated mice was unchanged relative to PBS (Fig. 2*F*), supporting increased thermogenesis rather than increased physical activity as the primary driver of elevated energy expenditure. Of note, locomotor activity was elevated in untargeted-treated mice relative to PBS (Fig. 2*F*), which may explain why energy expenditure was slightly elevated in this model relative to PBS (Fig. 2*D*). Notably, spikes in activity (Fig. 2*F*) align chronologically with spikes in oxygen consumption (Fig. 2*C*). Untargeted- and PBS-treated mice had a similar slope relating their body weight to energy expenditure (p-adj = 0.99) (Fig. 2*D*), further supporting that the untargeted control did not itself enhance thermogenesis. Respiratory exchange ratio (RER) remained unchanged, suggesting no major shift in whole-body substrate utilization on a chow diet after several weeks of treatment (Fig. 2*G*). *Zfp423* expression was reduced across brown, inguinal, and gonadal adipose depots relative to both controls, whereas *Ebf2* expression was unchanged (Fig. 2*H*), consistent with established models in which ZFP423 represses the thermogenic program by recruiting a corepressor complex to enhancer-bound EBF2 (24). In parallel, expression of downstream thermogenic and beiging markers, including *Ucp1*, was increased in *Zfp423* ASO-treated mice, but not in untargeted ASO-treated mice (Fig. 2*I*). Circadian clock gene expression remained unchanged across treatment conditions in inguinal white adipose tissue (iWAT), suggesting that observed time-dependent activation of thermogenesis was not due to altered adipose clock gene expression (*SI Appendix*, Fig. S2*A*). Overall, ASO37 showed a more consistent thermogenic response than ASO44, including smaller gonadal adipocytes; therefore, ASO37 was selected as the best candidate therapy design for subsequent studies. Collectively, these findings demonstrate that adipose-targeted ZFP423 suppression induces white adipose tissue beiging, enhances thermogenic energy expenditure, and improves glucose homeostasis in lean animals.

### *Zfp423* ASO Treatment Preserves Skeletal Muscle and Cardiac Function in Chow-Fed Mice.

Because *Zfp423* ASO treatment increased lean mass on a chow diet compared to controls (Fig. 1*D*), we next assessed skeletal muscle morphology and function. ZFP423 has been implicated in intramuscular adipogenesis (41–43), raising the possibility that ZFP423 suppression might alter skeletal muscle composition or function. Histologic analysis of gastrocnemius muscles showed normal muscle morphology, and grip strength testing revealed no measurable differences among treatment groups, indicating preserved skeletal muscle function even with reduced *Zfp423* expression in the muscle (*SI Appendix*, Fig. S2 *B*–*D*).

While heart rate increased following ASO37 treatment compared to PBS, no significant difference was observed between the ASO37 and untargeted treatments (*SI Appendix*, Fig. S2 *E* and *F*). Ejection fraction, E/A wave ratio, and left ventricular diameter in systole was unchanged across all groups by echocardiography (*SI Appendix*, Fig. S2 *G* and *H* and Movie S1 *A*–*C*). Together these findings demonstrate that *Zfp423* ASO treatment preserves both skeletal muscle and cardiac function in chow-fed mice.

### *Zfp423* ASO Promotes Dose-Dependent Metabolic Improvement and Enhances Mitochondrial Activation in Diet-Induced Obesity.

**Fig. 3.**
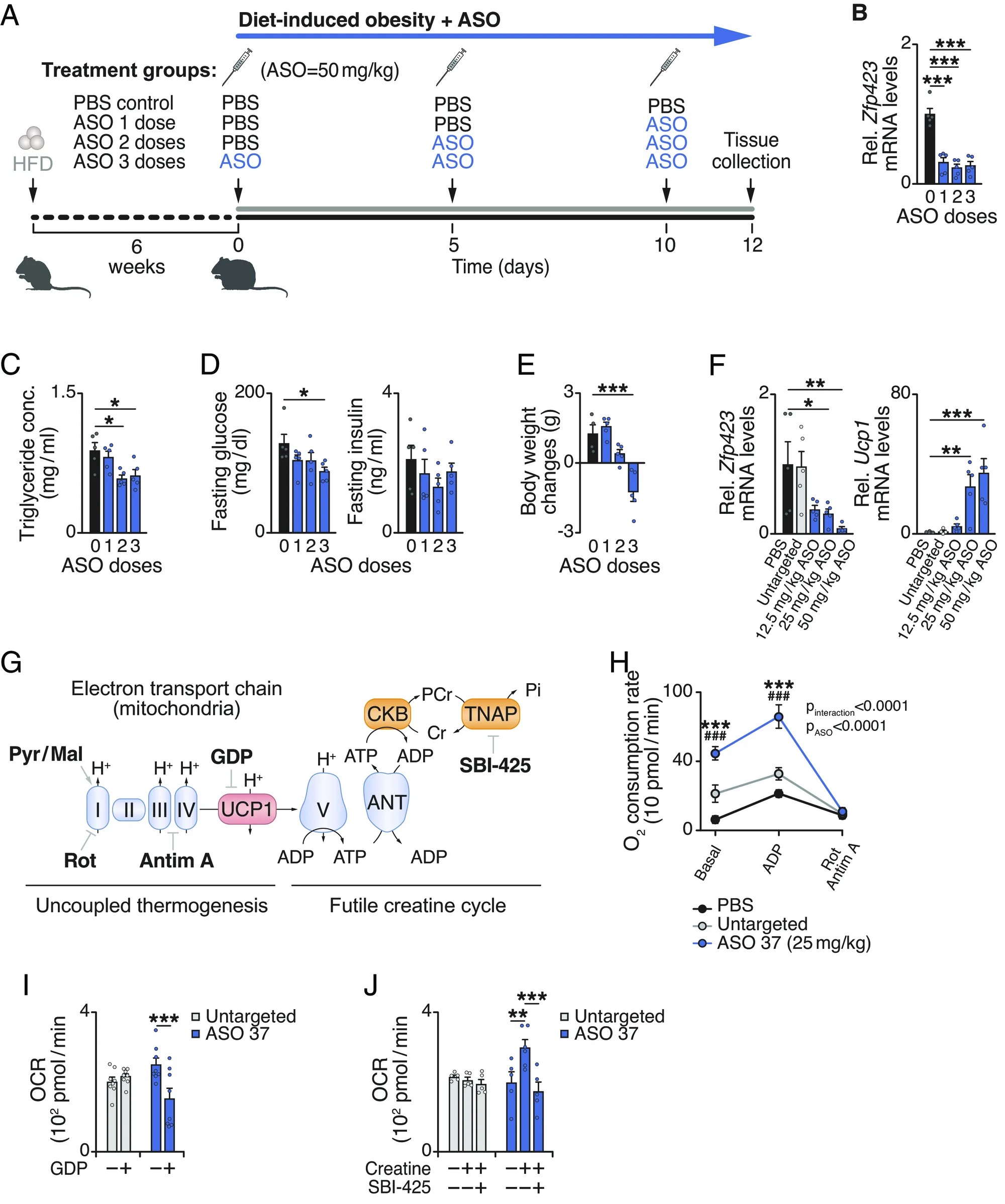
*Zfp423* ASO promotes dose-dependent metabolic improvement and enhances mitochondrial activation in diet-induced obesity. (*A*) Experimental schematic: Mice were fed a high-fat diet (HFD) for 6 wk before receiving PBS or ASO37 at equivalent volumes. Mice received 0-3 ASO doses (50 mg/kg/dose) over 10 d prior to tissue collection at ZT2. (*B*) Relative *Zfp423* mRNA levels, (*C*) fasting serum triglycerides, and (*D*) fasting blood glucose and insulin at tissue collection (n = 5). (*E*) Change in body weight from day 0 to day 12 (n = 5). (*F*) Relative *Zfp423* expression after three doses of ASO37 at indicated concentrations. (*G*) Mitochondrial electron transport chain schematic depicting compounds used in oxygen consumption rate (OCR) experiments; substrates were pyruvate and malate. (*H*) OCR of isolated mitochondria (5 μg/well) from HFD-fed mice treated with three doses of ASO37 or controls at 25 mg/kg/dose with indicated compounds (n = 5 mice, 10 technical replicates). * ASO37 vs PBS; # ASO37 vs untargeted. (*I*) OCR of isolated mitochondria (10 μg/well) from chow-fed mice treated with three doses of ASO37 or control with ADP ± GDP (n = 8 mice, 5 technical replicates). (*J*) Mitochondrial OCR as in panel I with limiting ADP and GDP. Creatine and SBI-425 were added as indicated (n = 8 mice, 8 technical replicates). Data are mean ± SEM. Statistics: two-way ANOVA with Tukey correction for *H*–*J*; otherwise, one-way ANOVA with Dunnett correction was performed. *P < 0.05, **P < 0.01, *** or ###*P* < 0.001.

To determine whether *Zfp423*-targeting ASOs confer metabolic benefits in the setting of established obesity, mice were first maintained on a high-fat diet for 6 wk and then treated with zero, one, two, or three doses of *Zfp423* ASO over 10 d before phenotyping (Fig. 3*A*). All groups received equivalent injection volumes at each treatment time point, using saline or ASO as appropriate, and tissues were harvested 2 d after the final injection (Fig. 3*A*). A single dose of ASO37 suppressed *Zfp423* expression by approximately 70% (Fig. 3*B*); however, measurable metabolic improvements required repeated dosing (Fig. 3 *C*–*E* and *SI Appendix*, Fig. S3*A*). Fasting serum triglyceride concentrations were significantly reduced after two doses of ASO37, whereas fasting glucose and body weight changes were reduced after three doses (Fig. 3 *C*–*E* and *SI Appendix*, Fig. S3*A*). Circulating nonesterified free fatty acids were unchanged (*SI Appendix*, Fig. S3*B*). These findings indicate that *Zfp423* knockdown from ASO treatment occurs prior to metabolic improvements. Lower ASO doses were also evaluated using the same dosing regimen in obese mice. The 50 mg/kg per dose still produced the greatest *Zfp423* suppression, achieving greater than 75% knockdown (92% efficiency) (Fig. 3*F*). Consistent with greater target suppression, *Ucp1* expression was maximally induced at 50 mg/kg compared with 25 or 12.5 mg/kg (Fig. 3*F*).

To determine whether the metabolic improvements following *Zfp423* knockdown were associated with enhanced adipose mitochondrial function, OCR was measured in isolated mitochondria from iWAT using pyruvate and malate as substrates under basal and ADP-stimulated conditions. Adipose tissue from ASO37-treated mice exhibited increased OCR compared with tissue from PBS (>threefold) and untargeted ASO controls (>twofold), consistent with increased mitochondrial oxidative capacity following *Zfp423* suppression (Fig. 3 *G* and *H*). Mitochondria isolated from iWAT of mice treated with untargeted ASO showed no significant decrease in OCR following the addition of guanosine diphosphate (GDP), an inhibitor of UCP1, in the presence of ADP, pyruvate, and malate (Fig. 3*I*). In contrast, mitochondria from mice treated with ASO37 showed a 39% decrease in OCR (*P* < 0.001), consistent with enhanced UCP1-dependent respiration (Fig. 3*I*). Similarly, when provided with limiting concentrations of ADP, mitochondria from mice treated with untargeted ASO did not increase their OCR in response to creatine, while mitochondria from ASO37-treated mice showed a 50% increase in OCR in response to creatine (*P* < 0.01) (Fig. 3*J*). This response was inhibited by SBI-425, an inhibitor of TNAP and futile creatine cycling (*P* < 0.001) (Fig. 3*J*).

Consistent with limited blood–brain barrier permeability of ASOs, *Zfp423* expression was unchanged in the thalamus and the hypothalamus, regions with high baseline *Zfp423* expression, after ASO treatment, supporting a peripheral mechanism of action (*SI Appendix*, Fig. S3*C*) (44). In diet-induced obese mice treated for 4 wk, heart rate remained unchanged between untargeted and ASO37 treatment groups (*SI Appendix*, Fig. S3 *D* and *E* and Movie S2*A* and *B*). Echocardiography done in the diet-induced obese condition (*SI Appendix*, Fig. S3*F*) showed impaired ejection fraction and left ventricular diameter in systole for untargeted controls, indicative of impaired cardiac function and hypertrophy (*P* < 0.05) (*SI Appendix*, Fig. S2*G* and S3 *F* and *G*). In contrast, ASO37 improved ejection fraction and left ventricular diameter in systole (*P* < 0.05) (*SI Appendix*, Fig. S3 *F* and *H*), consistent with a cardioprotective action in the setting of DIO. Collectively, these findings demonstrate that dose-dependent RNA therapy improves metabolic function, cardiac function, and adipose mitochondrial respiration in obesity.

### *Zfp423* ASO Enhances GLP-1RA-Mediated Weight Loss While Preserving Lean Mass.

**Fig. 4.**
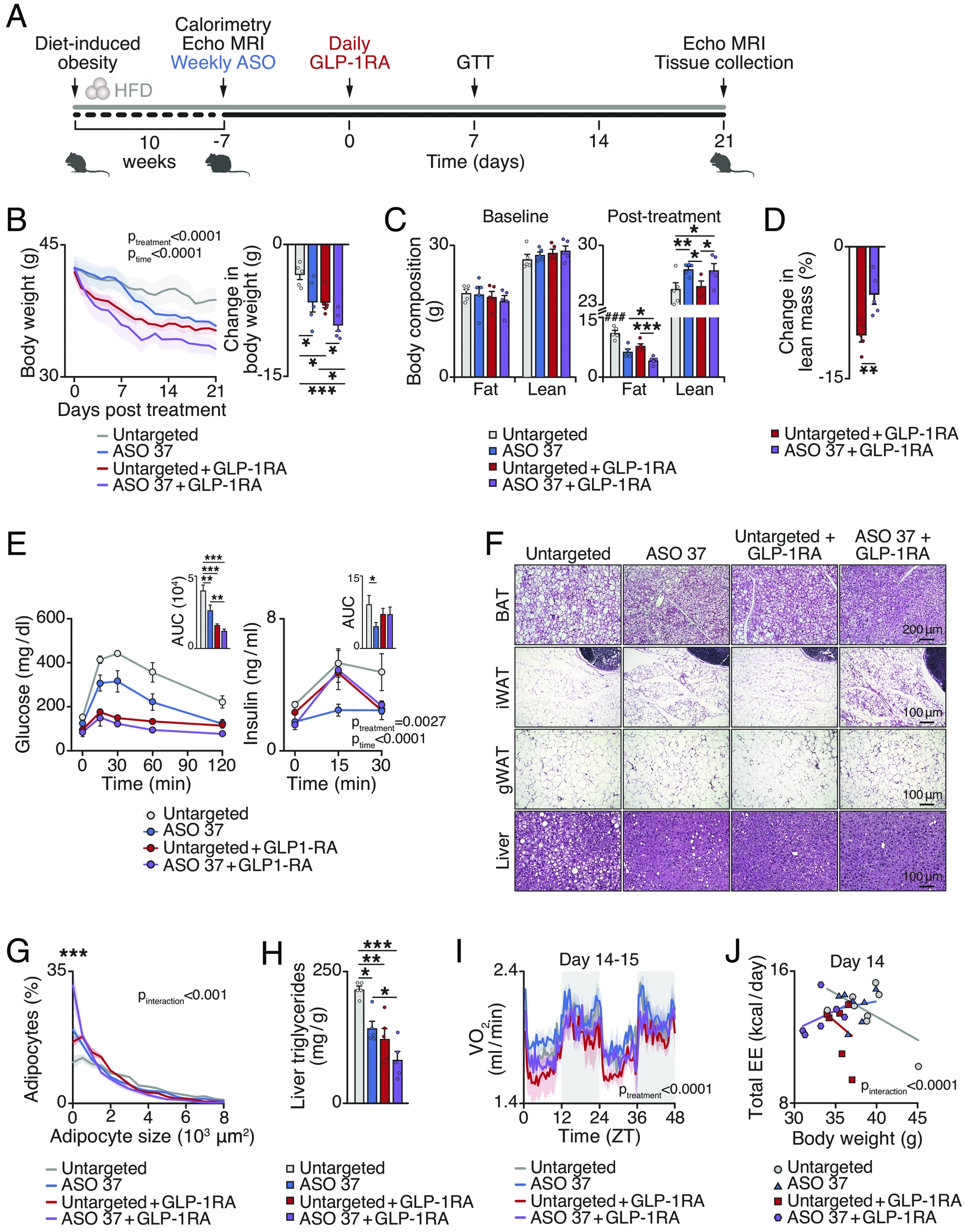
*Zfp423* ASO enhances GLP-1RA-mediated weight loss while preserving lean mass. (*A*) Experimental design: mice were fed HFD for 10 wk before dosing. Mice were acclimated to Sable metabolic phenotyping chambers and received ASO37 or untargeted ASO (50 mg/kg/wk) 1 wk before daily GLP-1RA (semaglutide; 3 nmol/kg/d) or PBS. Mice were matched by body weight and composition at day −7. (*B*) Body weight after initiation of daily GLP-1RA dosing. Change in body weight from day 0 to 21 (n = 5). (*C*) Body composition on day −7 (baseline) and day 21 (posttreatment); ###*P* < 0.001 relative to all treatment conditions for fat mass (n = 5). (*D*) Percent change in lean mass from baseline to day 21 (n = 5). (*E*) Glucose tolerance test (GTT) and insulin at ZT14 on day 7 (n = 5). (*F*) Representative hematoxylin and eosin (H&E) staining of BAT, iWAT, gWAT, and liver. (*G*) gWAT adipocyte size distribution at day 21 (n = 5). (*H*) Liver triglycerides. (*I*) Oxygen consumption after 2 wk of treatment; all pairwise comparisons *P* < 0.001 (n = 6 to 8). (*J*) 24-h energy expenditure vs body weight after 2 wk of GLP-1RA treatment, analyzed by Gaussian GLM as in Fig. 2. Relative to untargeted: P_ASO37_ < 0.0001, P_ASO37+GLP-1RA_ < 0.0001, P_GLP-1RA_ = 0.95 (ns) (n = 6 to 8). Data are mean ± SEM. Statistics: one- or two-way ANOVA with Tukey correction. *P < 0.05, **P < 0.01, ***P < 0.001

Because *Zfp423* ASO promoted fat loss with lean mass preservation (Fig. 1*D*), we next tested whether *Zfp423* suppression might complement GLP-1RA treatment, which is often associated with variable sarcopenia (45). Under chow-fed conditions, *Zfp423* ASO increased both food intake and energy expenditure in relation to body weight (Fig. 2 *B* and *D*), suggesting a shift toward increased energy turnover rather than reduced caloric intake. We therefore examined the effect of combining adipose thermogenic activation via ZFP423 inhibition with GLP-1RA-mediated appetite suppression in diet-induced obese mice. Mice were maintained on a HFD for 10 wk, followed by 1-wk acclimation in metabolic phenotyping chambers (Fig. 4*A*). Mice then received a 1-wk ASO pretreatment with either untargeted ASO or ASO37. Thereafter, mice were treated with daily subcutaneous semaglutide, 3 nmol/kg/dose at ZT10, or PBS vehicle, in combination with weekly injections of ASO37, 50 mg/kg/dose, or untargeted ASO (16) (Fig. 4*A* and *SI Appendix*, Fig. S4*A*).

GLP-1RA-treated mice lost weight more rapidly than ASO37-treated mice during the early treatment period; however, by the end of the study, body weight was similar between the two monotherapy groups. Combination therapy produced greater total weight loss (9.2 ± 0.7 g) than either intervention alone (6.6 ± 0.6 g for GLP-1RA and 6.6 ± 1.2 g for ASO37) (Fig. 4*B*). By study completion, body composition was also different. ASO37-treated mice had 6.4 ± 0.6 g body fat (*P* < 0.0001), while GLP-1RA-treated mice had 7.9 ± 0.5 g body fat (*P* < 0.0005) and combination therapy-treated mice had reduced their body fat to only 4.2 ± 0.4 g (*P* < 0.0001). Mice treated with ASO37 retained more of their lean mass in grams regardless of whether they received a GLP-1RA (*P* < 0.01) (Fig. 4*C*). GLP-1RA-treated mice lost approximately 10% (2.9 ± 0.3 g) of their starting lean mass, but combination-therapy-treated mice only lost 5.5% (1.6 ± 0.4 g) (p = 0.008 relative to GLP-1RA monotherapy) (Fig. 4*D*). Collectively, these results indicate that targeting *Zfp423* expression pharmacologically promotes significant lean mass preservation when used in combination with a GLP-1RA.

Glucose tolerance improved significantly with GLP-1RA, ASO37, and combination therapy relative to untargeted controls (*P* < 0.0001, 0.01, and 0.0001, respectively). ASO37 reduced insulin relative to untargeted control during glucose challenge, indicating improved insulin sensitivity (*P* < 0.01). Because *Zfp423* is not known to be expressed in insulin-producing cell types of the pancreas, this improvement is not likely due to a direct effect in the pancreas, but rather likely results from reduced adiposity and improved circulating lipids (Figs. 3*C* and 4*C*) (46, 47). GLP-1RA-treated mice retained insulin responsiveness 15 min after a glucose challenge and exhibited lower insulin levels 30 min after a glucose challenge (*P* < 0.05) (Fig. 4*E*). This effect is expected as GLP-1RAs directly trigger the pancreas to release insulin in a glucose-dependent manner (48). Adipose histology showed thermogenic browning/beiging in BAT and iWAT after ASO37 treatment (Fig. 4*F*). In gWAT, which does not readily beige, treatment did not induce a multilocular beige adipocyte phenotype. Instead, a greater percent of adipocytes were smaller than 500 um^3^ when treated with GLP-1RA or ASO37 relative to untargeted controls (both *P* < 0.0001), and even more adipocytes were smaller than 500 um^3^ following combination therapy (*P* < 0.0001 relative to both GLP-1RA and ASO37 monotherapies) (Fig. 4 *F* and *G*). GLP-1RA and ASO37 monotherapies each reduced hepatic triglyceride accumulation, liver lipid burden, liver weight, and liver-to-body weight ratio, with the greatest reductions after combination therapy (Fig. 4*H* and *SI Appendix*, Fig. S4*B*). iWAT depot weights and serum leptin were reduced more after dual therapy than GLP-1RA treatment alone (*P* < 0.01 and *P* < 0.05, respectively) (*SI Appendix*, Fig. S4 *C* and *D*). Quad weights relative to body weight were improved after dual therapy, and morphology was normal by histology (*P* < 0.01) (*SI Appendix*, Fig. S4 *E* and *F*). In obese mice, muscle function was improved for ASO37 monotherapy after 4 wk of ASO both before and after normalizing to body weight (*P* < 0.0001). Dual therapy mice also exhibited improved strength when controlled for body weight at the end of the dosing paradigm compared with mice that only received GLP-1RA (*P* < 0.01) (*SI Appendix*, Fig. S4*G*).

*Zfp423* expression levels were reduced to a similar extent in ASO37 monotherapy and dual therapy (*SI Appendix*, Fig. S4*H*). Beiging markers *Ucp1* and *Cidea* similarly increased between these two groups, suggesting that the entire beiging effect is due to ASO37 (Fig. S4*I*).

GLP-1RA treatment reduced food intake as expected (*P* < 0.05) (*SI Appendix*, Fig. S5 *A* and *B*). RER changes were most pronounced in the first 2 wk, and combination-treated mice had the lowest RER, indicating that the combination-therapy-treated mice were preferentially utilizing lipids as fuel (*SI Appendix*, Fig. S5*C*). Both ASO37 and GLP-1RA monotherapy treatments also resulted in reduced RER (*P* < 0.05) (*SI Appendix*, Fig. S5*C*). While GLP-1RA-treated mice showed 13% reduced oxygen consumption at 2 wk relative to controls (n = 7 to 8, *P* < 0.0001), combination therapy with ASO37 increased oxygen consumption by 4.8% relative to GLP-1RA monotherapy (n = 6 to 7, *P* < 0.0001) (Fig. 4*I*). Energy expenditure was still reduced relative to untargeted controls in GLP-1RA monotherapy (*P* < 0.0001) and combination therapy treatment conditions (*P* < 0.05) when assessed across the full 21-d treatment (Fig. 4*J* and *SI Appendix*, Fig. S5 *D* and *E*). Gaussian generalized linear modeling (GLM) indicates that both ASO37 and dual therapies increase the slope of the relationship between body weight and energy expenditure (*P* < 0.0001), while GLP-1RA-treated mice primarily maintain a lower energy expenditure without a concomitant shift in slope at days 1, 7, and 14 (Fig. 4*J* and *SI Appendix*, Fig. S5*E*). Locomotor activity during the dark period was reduced in mice treated with GLP-1RA (*P* < 0.05) and in mice treated with ASO37 (*P* < 0.05) (*SI Appendix*, Fig. S5*F*). Together, these findings establish that adjuvant therapy with *Zfp423* ASO and GLP-1RA therapy increases both loss of adiposity and preservation of lean-mass loss.

### ZFP423 Suppression Induces Prothermogenic Gene Expression in Beige Adipocytes.

**Fig. 5.**
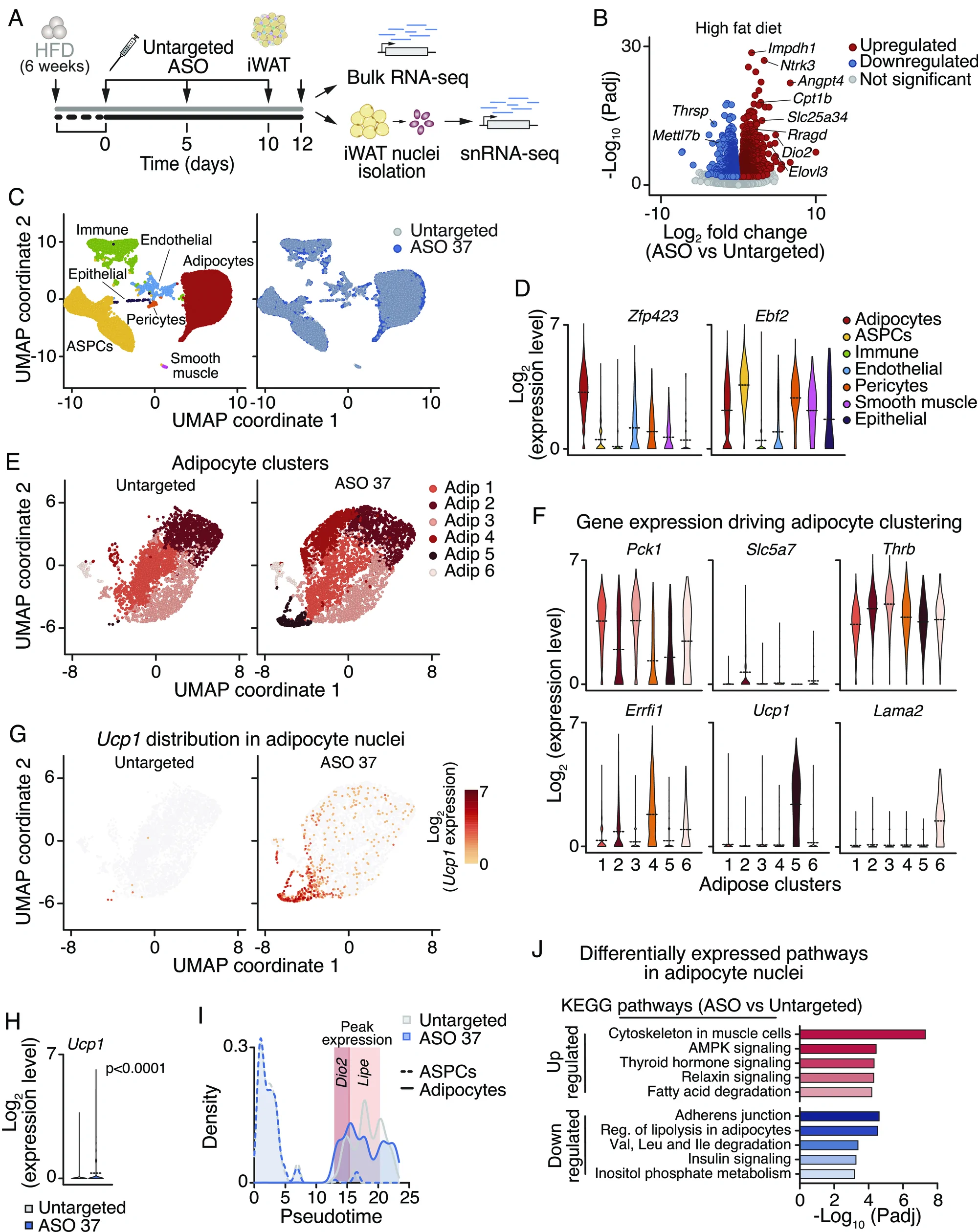
ZFP423 suppression induces prothermogenic gene expression in beige adipocytes. (*A*) Experimental schematic for single-nucleus RNA sequencing (snRNA-seq) and bulk RNA-seq studies. Wild-type diet-induced obese mice (6 wk HFD) were treated with an untargeted control ASO or ASO37 (three doses over 10 d), and tissues were collected 2 d after the final dose. Bulk RNA-seq was performed in parallel (n = 4/group). (*B*) Volcano plot of bulk RNA-sequencing comparing untargeted and ASO37-treated mice on HFD (n = 4/group). (*C*) Uniform manifold approximation and projection (UMAP) of iWAT nuclei from untargeted and ASO37-treated mice. *Left*: clustering by annotated cell type (adipocytes, adipose stromal and progenitor cells (ASPCs), endothelial, immune, epithelial, and smooth muscle cells). *Right*: clustering colored by treatment condition. (*D*) Violin plots of log2(expression) for *Zfp423* and *Ebf2* across annotated cell types. (*E*) UMAP of adipocyte nuclei by treatment condition, identifying 6 distinct adipocyte clusters (1 to 6). Clusters 4 and 5 are enriched in ASO37-treated samples. (*F*) Violin plots showing marker gene expression defining adipocyte clusters 1 to 6. (*G*) Dot plot and (*H*) violin plot showing *Ucp1* expression across adipocytes, comparing ASO treatment condition (Padj < 0.0001). (*I*) Pseudotime density plot for ASPCs and adipocytes overlaid with *Dio2* and *Lipe* expression (cutoff set to 0.02 and 2, respectively). ASO37 pseudotime density was increased in regions with high beige adipocyte marker gene expression. (*J*) Differentially expressed pathways in adipocyte nuclei, plotted against their respective −Log2(Padj). The dotted line represents the median for violin plots.

To define molecular pathways associated with the metabolic effects of *Zfp423* suppression, bulk RNA-sequencing was performed in the iWAT from chow-fed mice treated with ASO37 or a control ASO (*SI Appendix*, Fig. S6 *A* and *B*) (49). ASO37-induced transcripts were enriched for glucose and lipid metabolism pathways (Padj = 1.78e−4 and 4.22e−5, respectively), whereas downregulated transcripts were enriched for lipid biosynthesis programs (Padj = 3.49e−4) (*SI Appendix*, Fig. S6 *A* and *B*), consistent with reduced adiposity, improved glucose tolerance, and increased energy expenditure after *Zfp423* inhibition. ASO37-treated diet-induced obese mice showed >16-fold repression of *Zfp423* and induction of thermogenic and beige adipocyte programs, including increased expression of *Ucp1* and *Dio2* (Padj < 0.0001) (Fig. 5 *A* and *B*). Expression of genes involved in fatty acid transport and beta-oxidation, including *Elovl3* and *Cpt1b*, was also increased, consistent with enhanced lipid utilization in thermogenic adipose tissue (Padj < 0.0001) (50–52). Additional upregulated transcripts included genes linked to mitochondrial function and nutrient sensing, including *Slc25a34, Rragd,* and *Ntrk3* (Padj < 0.0001) (53–55), whereas downregulated genes included lipid storage and biosynthesis regulators such as *Thrsp* and *Mettl7b* (Padj < 0.0001). These transcriptional changes indicate a shift from lipid storage toward energy mobilization and adaptive metabolic remodeling.

Single-nucleus RNA-seq of iWAT from HFD-fed mice identified cell type–specific responses to ASO37 treatment (Fig. 5*A*) (56). After filtering and doublet removal, 17,067 nuclei were retained, including 7,707 adipocyte nuclei. Clusters were annotated using established marker genes (Fig. 5*C*) (57, 58). Most cell populations overlapped between groups, whereas adipocytes displayed a distinct ASO37-enriched cluster (Fig. 5 *C* and *D*). Reclustering identified six transcriptionally distinct adipocyte states, designated Adip-1 through Adip-6 (Fig. 5*E*). Adip-1 expressed white adipocyte markers, including *Pck1,* whereas Adip-2 expressed neuronal-associated genes such as *Slc5a7.* Adip-3 expressed *Thrb,* which receives signals from T3. Cluster Adip-4, enriched after ASO37-treatment, expressed *Errfi1*, a regulator of adaptive thermogenesis (59). Cluster Adip-5, also ASO-enriched, was defined by *Ucp1* expression, whereas Adip-6 expressed extracellular matrix-associated genes including *Lama2* (Fig. 5 *E*–*H*). UCP1-positive adipocytes were increased following ASO37 treatment, consistent with beige adipocyte expansion (Fig. 5 *G* and *H*). Pseudotime analysis demonstrated a transition from a mature white adipocyte (*Lipe*-expressing) state toward a *Dio2*-expressing beige adipocyte state (Pseudotime density differs where *Dio2* expression >0.02 and *Lipe* expression > 2.0) (Fig. 5*I*). KEGG pathway analysis identified AMPK signaling, thyroid hormone signaling, and fatty acid degradation as top upregulated pathways (Padj = 3.66e−5, 4.84e−5, and 6.49e−5, respectively) (Fig. 5*J*) (60). Cytoskeletal and extracellular matrix pathways were also induced, consistent with adipose tissue remodeling during weight loss (61), whereas insulin signaling and amino acid metabolism pathways were downregulated, suggesting altered substrate utilization (Padj = 5.51e−4 and 4.25e−4, respectively) (62, 63). Collectively, these findings indicate that ASO37 activates beige adipocyte transcriptional programs characterized by induction of thermogenic, β3-adrenergic, and mitochondrial oxidative pathways together with coordinated extracellular matrix remodeling.

### Distinct Adipocyte Populations Express *Ucp1* and Creatine Biosynthetic Programs.

RNA-seq from *Zfp423* ASO-treated mice showed increased expression of the creatine transporter *Slc6a8* (Padj < 0.01), consistent with recent studies showing that ZFP423 inhibition enhances diet-induced thermogenesis through the futile creatine cycle (FCC) (18, 64). Single-nucleus analysis identified distinct *Ucp1*- and *Atp5k*-positive adipocytes, the latter of which is a hallmark of FCC thermogenesis (65). Further, snRNA-seq revealed a total of 607 *Ucp1*-positive nuclei and 495 *Atp5k*-positive nuclei, with only 66 coexpressing both RNAs, consistent with recent analyses (*P* < 0.01 by Fisher’s exact test) (*SI Appendix*, Fig. S6 *C* and *D*) (66). Consistent with distinct FCC and UCP1 adipocyte subtypes, *Ucp1*-enriched Adip-5 cluster showed little or no *Gatm* or *Gamt* expression, whereas these creatine biosynthetic genes were expressed in all other adipocyte clusters (*SI Appendix*, Fig. S6*E*).

Because glycerol can activate creatine cycling, the fatty acid utilization program induced by *Zfp423* suppression may promote creatine-dependent thermogenesis through posttranscriptional mechanisms rather than direct transcriptional induction of creatine biosynthetic enzymes (67, 68). Consistent with this possibility, *Ckb* and *Alpl* were both detected in the dataset (64, 68, 69). Thus, *Zfp423* suppression induces distinct thermogenic adipocyte programs, including *Ucp1*-positive beige adipocytes and adipocytes with creatine-associated metabolic features.

In silico analysis of skeletal muscle single-cell RNA-seq data showed that *Zfp423* and *Ebf2* expression was enriched primarily in fibroblasts, fibro-adipocyte progenitors (FAPs), and pericytes, with weaker expression in muscle stem cells and myonuclei (*SI Appendix*, Fig. S6 *F* and *G*) (70). These data suggest that ZFP423 activity in muscle is concentrated in stromal and fibro-adipogenic compartments, consistent with a potential role in intramuscular adipose deposition.

## Discussion

We establish ZFP423 as a druggable target to enhance thermogenic energy expenditure and metabolic health in obesity. Using an adipose-directed ASO, we demonstrate that ZFP423 suppression remodels adipose tissue and increases whole-body energy expenditure in both lean and obese mice. In lean animals, ZFP423 inhibition improves glucose tolerance and reduces adiposity despite increased food intake, with robust inguinal adipose beiging, mitochondrial respiration, and dose-dependent *Ucp1* induction. In obese mice, *Zfp423* ASO reduces fasting glucose, triglycerides, and hepatic lipid accumulation while promoting weight loss comparable to GLP-1 receptor agonists such as semaglutide. Combining *Zfp423* ASO with semaglutide yields additive benefits: greater weight loss, reduced liver triglycerides, and fat mass reduction while preserving lean mass.

Mechanistically, ZFP423 functions as a gatekeeper of adipocyte identity by suppressing the thermogenic program through chromatin accessibility regulation via collaboration with EBF2. A central advance is establishing ZFP423 not merely as a brake on white adipose tissue beiging, but as a druggable regulator of adipose plasticity that drives enhanced energy expenditure.

ZFP423 suppression produces metabolically favorable weight loss. Unlike appetite-suppressive therapies that reduce fat and lean mass, *Zfp423*-targeting ASO preserves lean mass while reducing adiposity (71). This is clinically significant: GLP-1-based therapies reduce muscle mass in preclinical and clinical studies and can decouple muscle size from function, impairing force generation (72, 73). In contrast, *Zfp423* ASO-treated mice maintain or improve muscle strength, suggesting that increasing energy expenditure through adipose thermogenesis represents a complementary approach to obesity treatment.

While our study did not examine long-term metabolic dysfunction-associated steatotic liver disease models, we observed reduced hepatic lipid accumulation in obese mice, with further improvement when combined with GLP-1RA. Targeting adipose-specific gene regulatory pathways reduces visceral adiposity and ectopic fat in the liver. A remaining question is whether this may further enhance GLP-1RA protection against hepatic steatosis and inflammation, particularly by reducing fibrosis.

Our study has important limitations. We have not yet validated ZFP423 suppression in human adipocytes; the ASOs target mouse ZFP423, not human ZNF423. Therapeutically enhancing adipose thermogenesis has long been pursued yet limited by challenges achieving sustained, tissue-specific activation. Higher thermogenic activity associates with improved metabolic health independent of obesity, and individuals with active thermogenic adipose tissue show lower visceral adiposity (74). Adipose-targeting ASOs that suppress specialized adipose transcriptional repressors may overcome these challenges as therapeutics for metabolically healthier weight loss.

## Materials and Methods

### Animals and Diets.

All animal experiments were performed in accordance with procedures approved by Northwestern University’s Institutional Animal Care and Use Committee. C57BL/6J mice were purchased from Jackson Laboratories, group-housed, and maintained at room temperature (21 to 23 °C) with a 12-h light/dark cycle and free access to water and food. Mice were fed a regular chow diet unless otherwise indicated. For HFD experiments, 10-wk-old mice received ad libitum HFD (45% kcal from fat, Bio-Serv #F6635) for 6 or 10 wk as indicated.

### Indirect Calorimetry.

Activity was monitored in singly housed mice in metabolic cages (Promethion, Sable Systems International, NV). Mice were acclimated for 4 to 7 d with ad libitum access to food and water. Food intake, VO_2_, VCO_2_, RER (VCO_2_/VO_2_), and locomotor activity (beam breaks) were continuously monitored.

### Surface Body Temperature Monitoring.

Body temperature was monitored in custom-designed cages (Phenome Technologies, Inc.) equipped with miniature long-wave infrared (LWIR) thermal cameras mounted centrally on the cage ceiling to capture the entire mouse. Cameras were factory-calibrated and verified using a reference prior to experiments. Thermal images were acquired at 3-min intervals and transmitted to a dedicated server for processing using custom Python scripts. Raw images were converted to temperature maps and analyzed using standard image processing libraries to identify the mouse and extract temperature values. For each image, maximum surface temperature was used as a proxy for body temperature. Data were aggregated into 1-h bins, and continuous 48-h recordings were plotted for each animal.

### Metabolic Profiling.

To collect serum, tail blood was collected using Sarstedt Microvette tubes, placed on ice during collection, and separated by centrifugation. For glucose tolerance tests, mice were fasted for 6 h and administered glucose intraperitoneally at ZT14 (2 g/kg body weight, Sigma). Blood glucose was measured using Bayer Contour glucometers at the indicated timepoints. Serum insulin was measured using an Ultra-Sensitive Mouse Insulin ELISA (Crystal Chem). Terminal blood was collected by cardiac puncture. Leptin was measured in duplicate and averaged using the Milliplex Mouse Adipokine kit (Millipore Cat.#MADKMAG-71K), with plates read on Luminex 200 according to the recommended protocol.

### Body Composition Analysis.

Lean and fat mass were measured using EchoMRI-100 (EchoMRI, Houston, TX). Mice were weighed prior to analysis.

### Histological Analysis.

Adipose and liver tissue were harvested and incubated overnight (4 °C) in 4% paraformaldehyde and dehydrated in 70% ethanol. Muscle tissues were frozen, embedded, and cut before fixation and staining. Paraffin embedding, sectioning, and hematoxylin and eosin staining were performed by the Molecular Mouse Histology and Phenotyping Laboratory Core at Northwestern University. Images were obtained on a Keyence BZ-X810 (Japan). Adipocyte size was analyzed using Keyence Hybrid Cell Count software from brightfield images of H&E-stained sections. Quantification was performed by an investigator blinded to sample identity on >400 adipocytes per animal.

### Gene Expression Analysis by Quantitative PCR.

RNA isolation, cDNA synthesis, and quantitative PCR were performed as described (18). Adipose values were normalized to three housekeeping genes that remained steady in our models (*Actb, Rplp0, Ppia*). Otherwise, tissue-specific housekeeping genes were used (heart: *Ppia*, muscle: *Rpl13*, liver: *Rplp0*).

### Hepatic and Serum Lipid Measurement.

Hepatic triglycerides were isolated from 100 to 200 mg frozen liver using Folch extraction as described (75). All steps used glassware rather than plastic. Additional details can be found in the *SI Appendix*. For blood triglycerides, serum was collected from overnight-fasted animals. Triglycerides were measured using Infinity Triglycerides Reagent (Thermo Fisher Scientific, TR22421) according to the manufacturer’s instructions, with glycerol (Sigma, G7793-5 mL) as a standard. Liver triglycerides were normalized to liver weight prior to extraction. Nonesterified free fatty acids were measured from serum using Wako Chemicals’ NEFA-HR(2) assay according to the manufacturer’s instructions.

### Mitochondrial Isolation and Respiration.

To isolate mitochondria, fresh iWAT was minced and homogenized using a drill-operated Teflon pestle in ice-cold MSHE buffer as described (76). Protein concentration was determined using a Pierce BCA Protein Assay (ThermoFisher Scientific). Isolated mouse iWAT mitochondria were assayed for OCR using a Seahorse XFe96 Extracellular Flux Analyzer (Agilent Technologies). For OCR assays, mitochondria were adhered via centrifugation to Seahorse 96-well culture plates (5 to 10 μg protein/well). Respiratory substrates (10 mM pyruvate + 5 mM malate) and inhibitors (1 mM GDP, 0.01 mM creatine, 10 μM SBI-425) were diluted in MAS buffer and added to mitochondria as described (76). Substrate injections were 4 mM ADP (0.1 mM ADP for Fig. 3 *I* and *J*), 10 μM oligomycin, 10 μM FCCP, and 10 μM antimycin A.

### Bulk RNA Sequencing.

RNA was isolated from the whole iWAT adipocyte depot as for qPCR. RNA quality, library preparation, average library size, and concentration were determined or performed as described (76). Pooled libraries were single-end sequenced and analyzed as described (76).

### Single-Nucleus RNA-Seq.

Single nucleus RNA-seq was performed on iWAT with the lymph nodes removed to specifically study the adipose tissue environment. Nuclei were isolated from frozen iWAT (77), and nuclei from four mice per condition were pooled. Single nucle RNA-seq libraries were prepared using 10X Genomics Chromium GEM-X Single Cell 3’ v4 kit with loading of ~15,000 total nuclei per sample and paired-end sequenced on the Illumina NextSeq 2000 instrument.

Demultiplexing and read alignment were performed as described with integration using Harmony (76). Dimensionality reduction and differential gene expression between clusters were performed as described with nuclei identified using established markers prior to filtering and reclustering (see *SI Appendix* for specifics) (76). Plots were visualized in Loupe Browser using imported analysis from Seurat. KEGG pathway enrichment of differentially expressed genes between clusters was performed using clusterProfiler. Single-nucleus trajectory inference used the slingshot package integrated with a preprocessed Seurat object filtered for ASPCs and adipocytes. An initial start point was established using early ASPC markers, and an unbiased pseudotime trajectory inference was then performed on PCA embeddings using SlingPseudotime. Pseudotime density plots were generated using ggplot2.

### Statistical Analyses.

Data were analyzed using R (v4.3.2) for sequencing, temperature, and calorimetry data and Prism (v10.6.1) for all other statistical analyses. Calorimetry bar plots are presented as estimated marginal means ± SEM and were analyzed using mixed-effects maximum likelihood regression (*lmer*) using link functions with time of day, treatment and their interaction as fixed variables and animal and measurement date as random variables. Model fits were assessed using Akaike information criterion (AIC). Post hoc comparisons were performed using the emmeans R package with Tukey or Sidak corrections. Calorimetry data presented as line plots were analyzed in Prism as described. Results are presented as mean ± SEM unless otherwise stated. Statistical tests and significance thresholds are specified in figure legends.

## Supplementary Material

Appendix 01 (PDF)

Movie S1A. **Echocardiogram videos from mice treated with ASO for four weeks (A)** PBStreated (**B**) Untargeted ASO-treated (**C**) ASO37-treated.

Movie S1B. **Echocardiogram videos from mice treated with ASO for four weeks (A)** PBStreated (**B**) Untargeted ASO-treated (**C**) ASO37-treated.

Movie S1C. **Echocardiogram videos from mice treated with ASO for four weeks (A)** PBStreated (**B**) Untargeted ASO-treated (**C**) ASO37-treated.

Movie S2A. **Echocardiogram videos from diet-induced obese mice treated with ASO for four weeks (A)** Untargeted ASO-treated (**B**) ASO37-treated.

Movie S2B. **Echocardiogram videos from diet-induced obese mice treated with ASO for four weeks (A)** Untargeted ASO-treated (**B**) ASO37-treated.

## Data Availability

Sequencing datasets have been deposited in NCBI-GEO (GSE346709, GSE346803) (49, 56). All study data are included in the article and/or supporting information. Previously published data were used for this work (70).

## References

[1] Z. Liu, T. Xiao, H. Liu, Leptin signaling and its central role in energy homeostasis. Front. Neurosci. **17**, 1238528 (2023). 10.3389/fnins.2023.1238528 PMC10644276 38027481

[2] G. C. Major , Clinical significance of adaptive thermogenesis. Int. J. Obes. (Lond.) **31**, 204–212 (2007). 10.1038/sj.ijo.0803523 17260010

[3] S. Kajimura, B. M. Spiegelman, P. Seale, Brown and beige fat: Physiological roles beyond heat generation. Cell Metab. **22**, 546–559 (2015). 10.1016/j.cmet.2015.09.007 PMC4613812 26445512

[4] R. L. Smith , Metabolic flexibility as an adaptation to energy resources and requirements in health and disease. Endocr. Rev. **39**, 489–517 (2018). 10.1210/er.2017-00211 PMC6093334 29697773

[5] R. Lomonaco , Effect of adipose tissue insulin resistance on metabolic parameters and liver histology in obese patients with nonalcoholic fatty liver disease. Hepatology **55**, 1389–1397 (2012). 10.1002/hep.25539 22183689

[6] A. Boccatonda , From NAFLD to MAFLD: Definition, pathophysiological basis and cardiovascular implications. Biomedicines **11**, 883 (2023). 10.3390/biomedicines11030883 PMC10046146 36979861

[7] J. Quek , Global prevalence of non-alcoholic fatty liver disease and non-alcoholic steatohepatitis in the overweight and obese population: A systematic review and meta-analysis. Lancet Gastroenterol. Hepatol. **8**, 20–30 (2023). 10.1016/S2468-1253(22)00317-X 36400097

[8] P. L. Valenzuela , Obesity and the risk of cardiometabolic diseases. Nat. Rev. Cardiol. **20**, 475–494 (2023). 10.1038/s41569-023-00847-5 36927772

[9] B. Lauby-Secretan , Body fatness and cancer-viewpoint of the IARC working group. N. Engl. J. Med. **375**, 794–798 (2016). 10.1056/NEJMsr1606602 PMC6754861 27557308

[10] L. Caceres , Influence of weight loss and weight regain on adipose tissue inflammation. Arterioscler. Thromb. Vasc. Biol. **45**, 2155–2163 (2025). 10.1161/ATVBAHA.125.322196 PMC12525331 41036560

[11] S. N. Dankel , Weight cycling promotes fat gain and altered clock gene expression in adipose tissue in C57BL/6J mice. Am. J. Physiol. Endocrinol. Metab. **306**, E210–E224 (2014). 10.1152/ajpendo.00188.2013 24302006

[12] J. P. H. Wilding , Once-weekly semaglutide in adults with overweight or obesity. N. Engl. J. Med. **384**, 989–1002 (2021). 10.1056/NEJMoa2032183 33567185

[13] W. T. Garvey , Tirzepatide once weekly for the treatment of obesity in people with type 2 diabetes (SURMOUNT-2): A double-blind, randomised, multicentre, placebo-controlled, phase 3 trial. Lancet **402**, 613–626 (2023). 10.1016/S0140-6736(23)01200-X 37385275

[14] A. J. Sanyal , Phase 3 trial of semaglutide in metabolic dysfunction-associated steatohepatitis. N. Engl. J. Med. **392**, 2089–2099 (2025). 10.1056/NEJMoa2413258 40305708

[15] H. J. van Eyk , Liraglutide decreases energy expenditure and does not affect the fat fraction of supraclavicular brown adipose tissue in patients with type 2 diabetes. Nutr. Metab. Cardiovasc. Dis. **30**, 616–624 (2020). 10.1016/j.numecd.2019.12.005 32127340

[16] S. Gabery , Semaglutide lowers body weight in rodents via distributed neural pathways. JCI Insight **5**, e133429 (2020). 10.1172/jci.insight.133429 PMC7213778 32213703

[17] N. Beauregard , Energy balance and obesity: The emerging role of glucagon like peptide-1 receptor agonists. Curr. Opin. Clin. Nutr. Metab. Care **27**, 472–478 (2024). 10.1097/MCO.0000000000001064 39150432

[18] C. Hepler , Time-restricted feeding mitigates obesity through adipocyte thermogenesis. Science **378**, 276–284 (2022). 10.1126/science.abl8007 PMC10150371 36264811

[19] Y. Gong , Time-restricted feeding improves metabolic syndrome by activating thermogenesis in brown adipose tissue and reducing inflammatory markers. Front. Immunol. **16**, 1501850 (2025). 10.3389/fimmu.2025.1501850 PMC11802511 39925816

[20] N. Vujovic , Late isocaloric eating increases hunger, decreases energy expenditure, and modifies metabolic pathways in adults with overweight and obesity. Cell Metab. **34**, 1486–1498.e7 (2022). 10.1016/j.cmet.2022.09.007 PMC10184753 36198293

[21] R. K. Gupta , Zfp423 expression identifies committed preadipocytes and localizes to adipose endothelial and perivascular cells. Cell Metab. **15**, 230–239 (2012). 10.1016/j.cmet.2012.01.010 PMC3366493 22326224

[22] M. Shao , Fetal development of subcutaneous white adipose tissue is dependent on Zfp423. Mol. Metab. **6**, 111–124 (2017). 10.1016/j.molmet.2016.11.009 PMC5220400 28123942

[23] M. Shao , Zfp423 maintains white adipocyte identity through suppression of the beige cell thermogenic gene program. Cell Metab. **23**, 1167–1184 (2016). 10.1016/j.cmet.2016.04.023 PMC5091077 27238639

[24] M. Shao , ZFP423 controls EBF2 coactivator recruitment and PPARgamma occupancy to determine the thermogenic plasticity of adipocytes. Genes Dev. **35**, 1461–1474 (2021). 10.1101/gad.348780.121 PMC8559675 34620682

[25] X. Wang , EBF2 condensates enable adipose thermogenesis through ZFP423 sequestration and epigenetic remodeling. Nat. Commun. **17**, 5607 (2026). 10.1038/s41467-026-72233-x PMC13315918 42026085

[26] A. R. Angueira , Early B cell factor activity controls developmental and adaptive thermogenic gene programming in Adipocytes. Cell Rep. **30**, 2869–2878.e4 (2020). 10.1016/j.celrep.2020.02.023 PMC7079313 32130892

[27] C. Hepler , Directing visceral white adipocyte precursors to a thermogenic adipocyte fate improves insulin sensitivity in obese mice. eLife 6, e27669 (2017). 10.7554/eLife.27669 PMC5552276 28722653

[28] L. Vishvanath , Pdgfrbeta+ mural preadipocytes contribute to adipocyte hyperplasia induced by high-fat-diet feeding and prolonged cold exposure in adult mice. Cell Metab. **23**, 350–359 (2016). 10.1016/j.cmet.2015.10.018 PMC4749445 26626462

[29] L. Roth , Thyroid hormones are required for thermogenesis of beige adipocytes induced by Zfp423 inactivation. Cell Rep. **43**, 114987 (2024). 10.1016/j.celrep.2024.114987 39580797

[30] T. Becher , Brown adipose tissue is associated with cardiometabolic health. Nat. Med. **27**, 58–65 (2021). 10.1038/s41591-020-1126-7 PMC8461455 33398160

[31] A. M. Cypess, Reassessing human adipose tissue. N. Engl. J. Med. **386**, 768–779 (2022). 10.1056/NEJMra2032804 35196429

[32] A. E. O’Mara , Chronic mirabegron treatment increases human brown fat, HDL cholesterol, and insulin sensitivity. J. Clin. Invest. **130**, 2209–2219 (2020). 10.1172/JCI131126 PMC7190915 31961826

[33] F. Shamsi, C. H. Wang, Y. H. Tseng, The evolving view of thermogenic adipocytes—Ontogeny, niche and function. Nat. Rev. Endocrinol. **17**, 726–744 (2021). 10.1038/s41574-021-00562-6 PMC8814904 34625737

[34] A. E. Chappell , Mechanisms of palmitic acid-conjugated antisense oligonucleotide distribution in mice. Nucleic Acids Res. **48**, 4382–4395 (2020). 10.1093/nar/gkaa164 PMC7192618 32182359

[35] T. P. Prakash , Fatty acid conjugation enhances potency of antisense oligonucleotides in muscle. Nucleic Acids Res. **47**, 6029–6044 (2019). 10.1093/nar/gkz354 PMC6614804 31127296

[36] J. Himms-Hagen , Multilocular fat cells in WAT of CL-316243-treated rats derive directly from white adipocytes. Am. J. Physiol. Cell Physiol. **279**, C670–C681 (2000). 10.1152/ajpcell.2000.279.3.C670 10942717

[37] N. Petrovic , Chronic peroxisome proliferator-activated receptor gamma (PPARgamma) activation of epididymally derived white adipocyte cultures reveals a population of thermogenically competent, UCP1-containing adipocytes molecularly distinct from classic brown adipocytes. J. Biol. Chem. **285**, 7153–7164 (2010). 10.1074/jbc.M109.053942 PMC2844165 20028987

[38] J. Ishibashi, P. Seale, Medicine. Beige can be slimming. Science **328**, 1113–1114 (2010). 10.1126/science.1190816 PMC2907667 20448151

[39] S. D. Parlee , Quantifying size and number of adipocytes in adipose tissue. Methods Enzymol. **537**, 93–122 (2014). 10.1016/B978-0-12-411619-1.00006-9 PMC4069255 24480343

[40] K. M. McCabe , Antisense oligonucleotide treatment produces a type I interferon response that protects against diet-induced obesity. Mol. Metab. **34**, 146–156 (2020). 10.1016/j.molmet.2020.01.010 PMC7036698 32180554

[41] Y. Huang , Zfp423 promotes adipogenic differentiation of bovine stromal vascular cells. PLoS One **7**, e47496 (2012). 10.1371/journal.pone.0047496 PMC3468566 23071815

[42] D. Q. Peng, S. B. Smith, H. G. Lee, Vitamin A regulates intramuscular adipose tissue and muscle development: Promoting high-quality beef production. J. Anim. Sci. Biotechnol. **12**, 34 (2021). 10.1186/s40104-021-00558-2 PMC7934359 33663602

[43] H. Rahemi, N. Nigam, J. M. Wakeling, The effect of intramuscular fat on skeletal muscle mechanics: Implications for the elderly and obese. J. R. Soc. Interface **12**, 20150365 (2015). 10.1098/rsif.2015.0365 PMC4535407 26156300

[44] M. Uhlen , Proteomics. Tissue-based map of the human proteome. Science **347**, 1260419 (2015). 10.1126/science.1260419 25613900

[45] J. Linge, A. L. Birkenfeld, I. J. Neeland, Muscle mass and glucagon-like peptide-1 receptor agonists: Adaptive or maladaptive response to weight loss? Circulation **150**, 1288–1298 (2024). 10.1161/CIRCULATIONAHA.124.067676 39401279

[46] M. Karlsson , A single-cell type transcriptomics map of human tissues. Sci. Adv. **7**, eabh2169 (2021). 10.1126/sciadv.abh2169 PMC8318366 34321199

[47] K. Hrovatin , Delineating mouse beta-cell identity during lifetime and in diabetes with a single cell atlas. Nat. Metab. **5**, 1615–1637 (2023). 10.1038/s42255-023-00876-x PMC10513934 37697055

[48] A. R. Meloni , GLP-1 receptor activated insulin secretion from pancreatic beta-cells: Mechanism and glucose dependence. Diabetes Obes. Metab. **15**, 15–27 (2013). 10.1111/j.1463-1326.2012.01663.x PMC3556522 22776039

[49] A. Thorne, J. Bass, Data from “An RNA thermogenic therapy to preserve lean mass and enhance metabolic health during GLP-1 weight loss.” Gene Expression Omnibus (GEO). https://www.ncbi.nlm.nih.gov/geo/query/acc.cgi?acc=GSE346709. Deposited 9 September 2026. 10.1073/pnas.2618845123 PMC13643317 42789307

[50] Z. Qin , ELOVL3 regulates phospholipid homeostasis and thermogenesis in brown adipose tissue. J. Lipid. Res. **66**, 100937 (2025). 10.1016/j.jlr.2025.100937 PMC12704387 41202879

[51] L. Vergnes , Heart-type fatty acid-binding protein is essential for efficient brown adipose tissue fatty acid oxidation and cold tolerance. J. Biol. Chem. **286**, 380–390 (2011). 10.1074/jbc.M110.184754 PMC3012996 21044951

[52] G. Wade , Lipid transport in brown adipocyte thermogenesis. Front. Physiol. **12**, 787535 (2021). 10.3389/fphys.2021.787535 PMC8733649 35002769

[53] I. Karavaeva , Mitochondrial carrier SLC25A34 links clock, diet, and temperature control of interorganellar lipid cycling. bioRxiv [Preprint] 2026). 10.64898/2026.05.30.724257 (Accessed 19 June 2026). 42821689

[54] E. Shapira , Butyrate-mediated upregulation of insulin pathway gene expression suggests potential antidiabetic effects. Drug Dev. Res. **86**, e70203 (2025). 10.1002/ddr.70203 PMC12665645 41320781

[55] M. Bove , NT3/TrkC pathway modulates the expression of UCP-1 and adipocyte size in human and rodent adipose tissue. Front. Endocrinol. (Lausanne) **12**, 630097 (2021). 10.3389/fendo.2021.630097 PMC8015941 33815288

[56] A. Thorne, J. Bass, Data from “An RNA thermogenic therapy to preserve lean mass and enhance metabolic health during GLP-1 weight loss.” Gene Expression Omnibus (GEO). https://www.ncbi.nlm.nih.gov/geo/query/acc.cgi?acc=GSE346803. Deposited 9 September 2026. 10.1073/pnas.2618845123 PMC13643317 42789307

[57] M. P. Emont , A single-cell atlas of human and mouse white adipose tissue. Nature **603**, 926–933 (2022). 10.1038/s41586-022-04518-2 PMC9504827 35296864

[58] Y. Wu , Dynamics of single-nuclei transcriptomic profiling of adipose tissue from diverse anatomical locations during mouse aging process. Sci. Rep. **14**, 16093 (2024). 10.1038/s41598-024-66918-w PMC11245496 38997312

[59] S. Choung , Mig-6 is essential for glucose homeostasis and thermogenesis in brown adipose tissue. Biochem. Biophys. Res. Commun. **572**, 92–97 (2021). 10.1016/j.bbrc.2021.07.088 34358969

[60] L. Wu , AMP-activated protein kinase (AMPK) regulates energy metabolism through modulating thermogenesis in adipose tissue. Front. Physiol. **9**, 122 (2018). 10.3389/fphys.2018.00122 PMC5826329 29515462

[61] I. V. de Sousa Neto , Adipose tissue extracellular matrix remodeling in response to dietary patterns and exercise: Molecular landscape, mechanistic insights, and therapeutic approaches. Biology (Basel) **11**, 765 (2022). 10.3390/biology11050765 PMC9138682 35625493

[62] A. R. P. Verkerke , BCAA-nitrogen flux in brown fat controls metabolic health independent of thermogenesis. Cell **187**, 2359–2374.e18 (2024). 10.1016/j.cell.2024.03.030 PMC11145561 38653240

[63] J. E. R. Grothen , Single cell transcriptomics of human weight loss links adipocyte NPY1R to control of lipolysis. Mol. Metab. **105**, 102305 (2026). 10.1016/j.molmet.2025.102305 PMC12926987 41412283

[64] L. Kazak , A creatine-driven substrate cycle enhances energy expenditure and thermogenesis in beige fat. Cell **163**, 643–655 (2015). 10.1016/j.cell.2015.09.035 PMC4656041 26496606

[65] T. Wang , Single-nucleus transcriptomics identifies separate classes of UCP1 and futile cycle adipocytes. Cell Metab. **36**, 2130–2145.e7 (2024). 10.1016/j.cmet.2024.07.005 39084216

[66] A. Vargas-Castillo , Development of a functional beige fat cell line uncovers independent subclasses of cells expressing UCP1 and the futile creatine cycle. Cell Metab. **36**, 2146–2155.e5 (2024). 10.1016/j.cmet.2024.07.002 PMC12005060 39084217

[67] M. F. Hussain , Glycerol-driven TNAP activation in thermogenesis and mineralization. Nature **654**, 198–208 (2026). 10.1038/s41586-026-10396-9 42020733

[68] Y. Sun , Mitochondrial TNAP controls thermogenesis by hydrolysis of phosphocreatine. Nature **593**, 580–585 (2021). 10.1038/s41586-021-03533-z PMC8287965 33981039

[69] J. F. Rahbani , Creatine kinase B controls futile creatine cycling in thermogenic fat. Nature **590**, 480–485 (2021). 10.1038/s41586-021-03221-y PMC8647628 33597756

[70] P. Zhu , Immunomodulatory role of the stem cell circadian clock in muscle repair. Sci. Adv. **11**, eadq8538 (2025). 10.1126/sciadv.adq8538 PMC11881903 40043110

[71] M. Alissou , Impact of Semaglutide on fat mass, lean mass and muscle function in patients with obesity: The SEMALEAN study. Diabetes Obes. Metab. **28**, 112–121 (2026). 10.1111/dom.70141 PMC12673431 41068996

[72] S. Jeromson , Semaglutide impacts skeletal muscle to a similar extent as caloric restriction in mice with diet-induced obesity. J. Physiol. JP289449 (2025). 10.1113/JP289449 40982727

[73] T. Karasawa , Unexpected effects of semaglutide on skeletal muscle mass and force-generating capacity in mice. Cell Metab. **37**, 1619–1620 (2025). 10.1016/j.cmet.2025.07.004 40769122

[74] C. T. Herz , Active brown adipose tissue is associated with a healthier metabolic phenotype in obesity. Diabetes **71**, 93–103 (2021). 10.2337/db21-0475 34957487

[75] J. P. Rose , FGF21 reverses MASH through coordinated actions on the CNS and liver. Cell Metab. **37**, 1515–1529.e6 (2025). 10.1016/j.cmet.2025.04.014 PMC12409791 40367940

[76] C. Hepler , Adipocyte NADH dehydrogenase reverses circadian and diet-induced metabolic syndrome. Nat. Metab. **8**, 559–571 (2026). 10.1038/s42255-026-01464-5 PMC13031130 41708974

[77] E. L. Van Hauwaert , Isolation of nuclei from mouse white adipose tissues for single-nucleus genomics. STAR Protoc. **2**, 100612 (2021). 10.1016/j.xpro.2021.100612 PMC8220393 34189477

