# Supplementary material for: An RNA thermogenic therapy to preserve lean mass and enhance metabolic health during GLP-1 weight loss
Source: Proc Natl Acad Sci U S A. 2026 Sep 25;123(40):e2618845123. doi: 10.1073/pnas.2618845123 (PMC13643317; doi:10.1073/pnas.2618845123)
Supplement: Supplementary file 1 [file pnas.2618845123.sapp.pdf]

PNAS

Supporting Information for
An RNA Thermogenic Therapy to Preserve Lean Mass and Enhance Metabolic Health During GLP-1 Weight Loss

Anneke K. Thorne[1], Nathan J. Waldeck[1], Jesse P. Rose[1], Marisa J. Stachowski[2], Hannah Guak[1], Alexander R. Keeble[2], Britta Kuusik[1], Ella Simon[1], Biliana Marcheva[1], Joseph V. Mastroni[1], Pei Zhu[3], Ling Wa Chong[4], Yumiko Kobayashi[1], Nicholas J. Conley[1], Andrew B. Cohen[1], Yasuhiro Omura[1], Chiaki Omura[1], Weimin Song[1], Michael Wellems[5], Kathryn M. Ramsey[1], Grant D. Barish[1], Lisa R. Beutler[1], Rana K. Gupta[6], Clara B. Peek[3], Elizabeth M. McNally[2], Chelsea Hepler[7], Mark Perelis[4], Joseph Bass[1]

[1]Department of Medicine, Division of Endocrinology, Metabolism, and Molecular Medicine, Feinberg School of Medicine, Northwestern University, Chicago, IL, 60611.
[2]Center for Genetic Medicine, Feinberg School of Medicine, Northwestern University, Chicago, IL, 60611.
[3]Department of Biochemistry and Molecular Genetics, Feinberg School of Medicine, Northwestern University, Chicago, IL, 60611.
[4]Ionis Pharmaceuticals, Carlsbad, CA, 92010.
[5]Phenome Technologies Inc., Lincolnshire, IL, 60091.
[6]Division of Endocrinology, Department of Medicine, Duke Molecular Physiology Institute, Duke University, Durham, NC, 27701.
[7]Department of Molecular & Integrative Physiology, University of Michigan, Ann Arbor, MI, 48109.
*Corresponding author: Joseph Bass

Email: j-bass@northwestern.edu

This PDF file includes:

Supporting Text
Figures S1 to S6
Tables S1 to S2
Legends for Movies S1 to S2
SI References

Other supporting materials for this manuscript include the following:

Movies S1 to S2

Supporting Information Text
Supplementary Methods

Hepatic and serum lipid measurement
For Folch extraction, mouse liver tissue was homogenized in 2:1 v/v chloroform/methanol and incubated at room temperature. 50mM NaCl was added to homogenates and vortexed for 15 seconds before centrifugation for 10 min at 1000 xg. The bottom organic phase was collected and mixed with 0.36 M $CaCl_2$/Methanol/H_20 (1:1 v/v Methanol/H_2O), vortexed, centrifuged as before, and the organic phase was collected again. The extract was then dried under nitrogen gas and resuspended in 1 mL isopropanol before measuring concentration.

Hepatic and kidney function tests
Measurements of hepatic function (AST, ALT, albumin, billirubin) and kidney function (creatinine, blood urea nitrogen) were run on a clinical chemistry analyzer on plasma samples (AU480, Beckman Coulter). Blood was collected at necropsy via cardiac puncture, and samples were separated by centrifugation in EDTA-coated tubes.

Cardiac function
All cardiac measurements and analyses were conducted blinded to treatment groups. For echocardiography, mice were anesthetized under 4% isoflurane and maintained under 1% throughout the procedure. Transthoracic echocardiography was performed using a Vevo 2100 (Fujifilm-Visualsonics) and MS400 transducer [1]. Short-axis M-mode imaging was used to measure left ventricular chamber dimensions. Left ventricular diastolic function was assessed using Doppler echocardiography from an apical-four chamber view. For electrocardiography (ECG), ECG was conducted in conscious mice using the ECGenie platform (Mouse Specifics, Inc, Framingham, MA) [2]. Mice were acclimatized to the platform for 10 minutes, followed by 15 minutes of data collection. Traces were analyzed using EzCG Signal Analysis Software.

Muscle strength analysis
Muscle strength measurements were conducted blinded to treatment groups. Grip strength was assessed using a grip strength meter set to measure peak force generated (Chatillon) according to standardized protocols [3]. Both a forelimb-specific setup and a fore- and hindlimb combined setup were utilized. Briefly, mice were allowed to contact a horizonal grid with the specified limbs, then were gently pulled backward horizontally until release. Peak force was recorded and the mean of 5 trials was plotted for each mouse with resting time between each trial. Grip strength was performed on chow-fed mice to avoid body weight as a confounder and was plotted as absolute grip strength. Additional muscle strength assessments were done targeting peak plantar complex tetanic isometric torque using a Whole Mouse Test system (1300A; Aurora Scientific, ON, Canada) equipped with a 1 N footplate force transducer (300C-LR; Aurora Scientific). Mice were maintained under deep anesthesia (2% isoflurane) on a heated platform throughout the assessment. The knee was securely clamped and the foot positioned on a footplate fixed

at 90º relative to the tibia. Bipolar silver electrodes were inserted subcutaneously over the tibial nerve, and muscle contractions were evoked using a 0.1 ms pulse width and 0.3 s train duration. A force-frequency curve was constructed by applying a series of stimulation frequencies (10, 40, 80, 125, and 200 Hz), with 30s of rest between contractions. Peak tetanic torque occurred at 125 Hz, and results were plotted both raw and normalized to body weight [4, 5].

Single nucleus RNA-seq

For sample preparation, iWAT was minced in nucleus preparation buffer (NPB; 10 mM HEPES, 1.5 mM MgCl2, 10 mM KCl, 250 mM sucrose, 0.001% NP-40) with 0.2 U/ul Roche Protector RNase inhibitor followed by dounce homogenization. The homogenate was filtered through a 100 uM cell strainer and centrifuged at 1000 xg for 10 minutes at 4°C, then the nuclear pellet was washed once in NPB followed by resuspension in PBS with 1% BSA and 0.2 U/ul Roche Protector RNase inhibitor.

Adipocyte nuclei were identified based on the expression of established markers (*Adipoq, Lipe, Plin4, Plin1*, and *Pparg*) and filtered for re-clustering using Seurat. Non-adipocyte nuclei were identified using established markers (adipocyte stromal and progenitor cells (ASPCs) with Pdgfra and Dpp4; endothelial cells with *Cdh5, Jam2* and *Prox1*; immune cell populations with *Foxp3, Adgre1, Cd14, Itgax, Kit, Itgam, Ncr1* and *Klrb1c*; epithelial cells with *Cdh1*; pericytes with *Pdgfrb* and *Mcam*; and smooth muscle cells with *Myh11*).

Cell Ranger software was used to perform demultiplexing and align reads to mm39. Nuclei were filtered using Seurat based on the total number of genes and molecules, as well as percent of mitochondrial reads (500 < nFeature_RNA < 6000; 1000 < nCount_RNA < 25000; percent.mt < 15%). Conditions were integrated using Harmony and dimensionality reduction was performed in Seurat using Uniform Manifold Approximation and Projection (UMAP). Differential gene expression between clusters was performed using FindMarkers function. One cluster lacking canonical cell-type markers was excluded. Adipocyte nuclei were identified based on the expression of established markers (*Adipoq, Lipe, Plin4, Plin1*, and *Pparg*) and filtered for re-clustering using Seurat. Non-adipocyte nuclei were identified using established markers (adipocyte stromal and progenitor cells (ASPCs) with Pdgfra and Dpp4; endothelial cells with *Cdh5, Jam2* and *Prox1*; immune cell populations with *Foxp3, Adgre1, Cd14, Itgax, Kit, Itgam, Ncr1* and *Klrb1c*; epithelial cells with *Cdh1*; pericytes with *Pdgfrb* and *Mcam*; and smooth muscle cells with *Myh11*). UMAP and violin plots of the adipocyte nuclei were visualized using Loupe Browser of the imported analysis from Seurat. KEGG pathway enrichment analysis of differentially expressed genes between clusters of samples was performed using clusterProfiler. Single-nucleus trajectory inference was performed using the slingshot package integrated with a preprocessed Seurat object filtered for ASPCs and adipocytes. An initial start point was established using early ASPC markers *Dpp4* and *Cd55* to distinguish from more committed precursors. An unbiased pseudotime trajectory inference was then performed on PCA embeddings using SlingPseudotime. Pseudotime density plots were then generated using ggplot2.

A

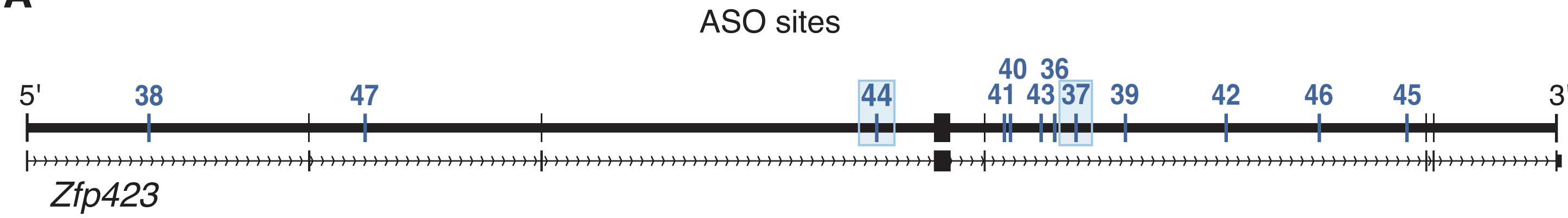

B

ASO	ASO equivalent sequence	pre-mRNA targeting sequence
Untargeted	5'-CGCCGATAAGGTACAC-3'	N/A
ASO 36	5'-AATGTTTAACGAGTCA-3'	5'-UGACUCGUUAAACAUU-3'
**ASO 37**	**5'-TTTTTATTACAGCGGA-3'**	**5'-UCCGCUGUAAUAAAAA-3'**
ASO 38	5'-AGAGATAATTCAGCGG-3'	5'-CCGCUGAAUUAUCUCU-3'
ASO 39	5'-GACATTAAGGTACTGC-3'	5'-GCAGUACCUUAAUGUC-3'
ASO 40	5'-ATTTTAGCGAGGTTCG-3'	5'-CGAACCUCGCUAAAAU-3'
ASO 41	5'-GGTATTAGAACAATTC-3'	5'-GAAUUGUUCUAAUACC-3'
ASO 42	5'-TCTGATAAGGCTTGGC-3'	5'-GCCAAGCCUUAUCAGA-3'
ASO 43	5'-AAGGATACAAGACTGG-3'	5'-CCAGUCUUGUAUCCUU-3'
**ASO 44**	**5'-ACCATAAGATTCACCA-3'**	**5'-UGGUGAAUCUUAUGGU-3'**
ASO 45	5'-ACTGATTTAACACACT-3'	5'-AGUGUGUUAAAUCAGU-3'
ASO 46	5'-CATAAATCTGATGCGG-3'	5'-CCGCAUCAGAUUUAUG-3'
ASO 47	5'-CCAGAATTGATACCCA-3'	5'-UGGGUAUCAAUUCUGG-3'

C

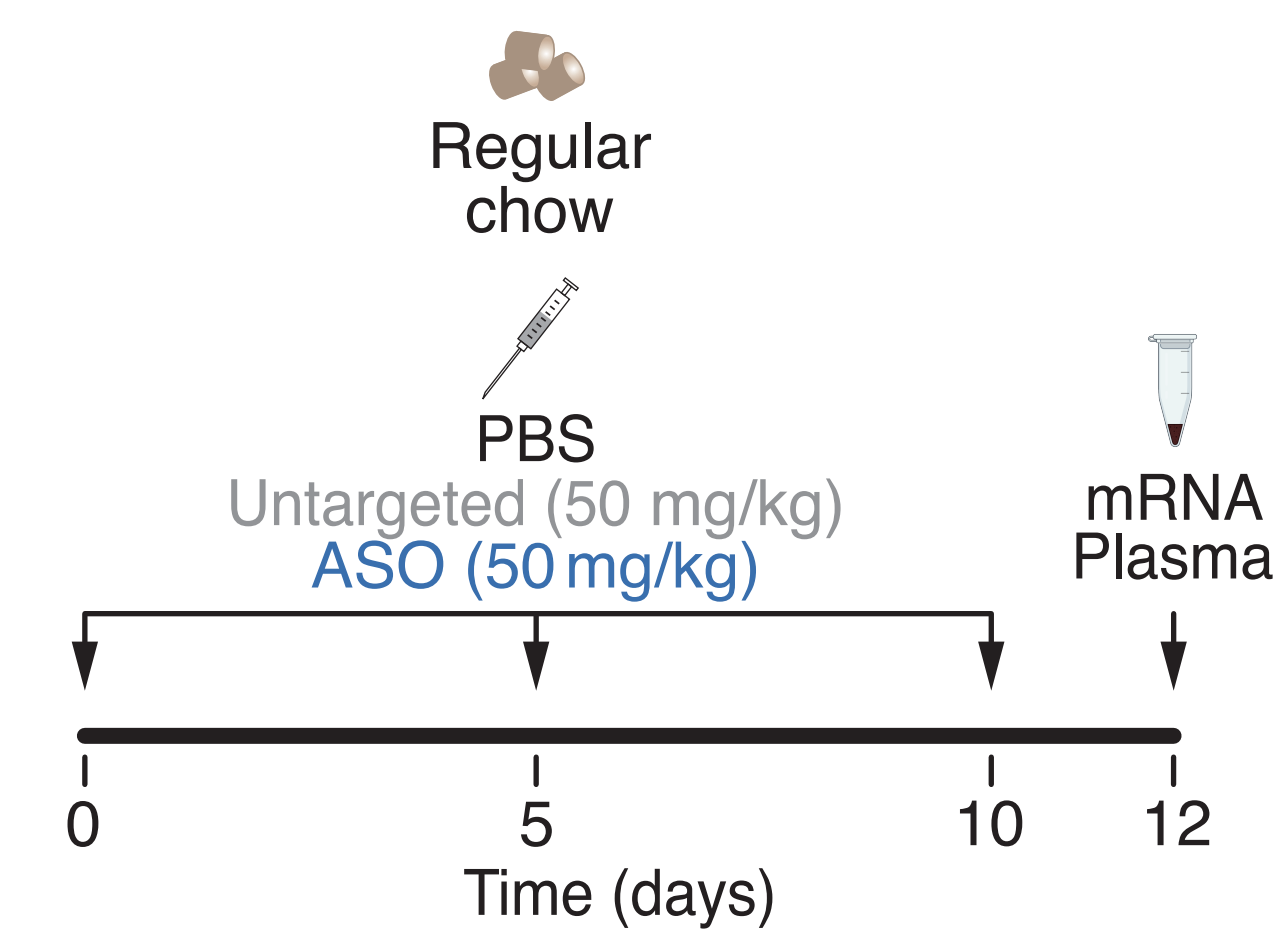

D

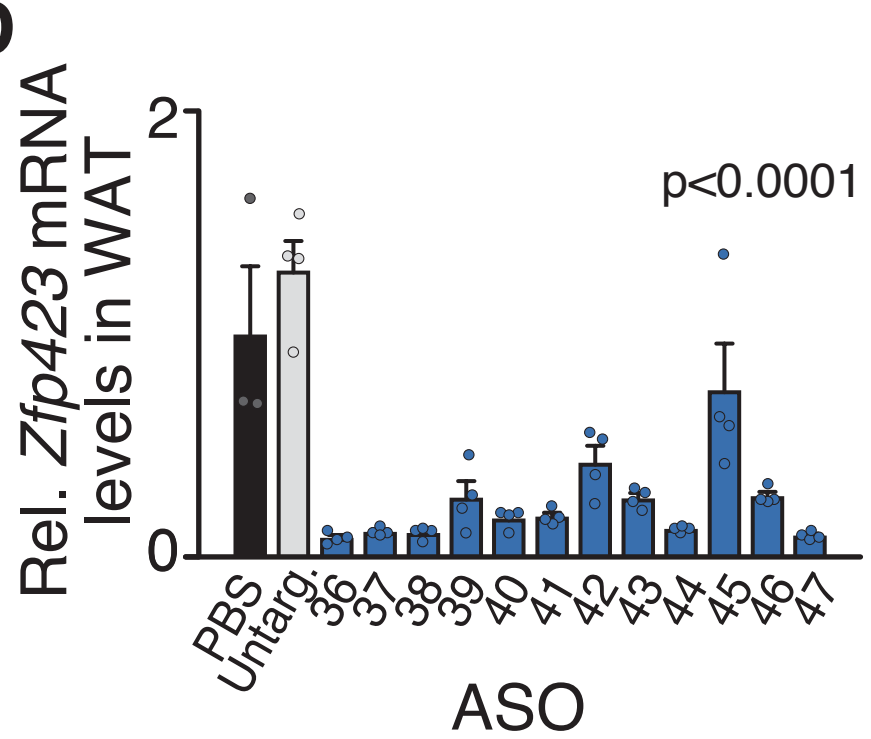

E

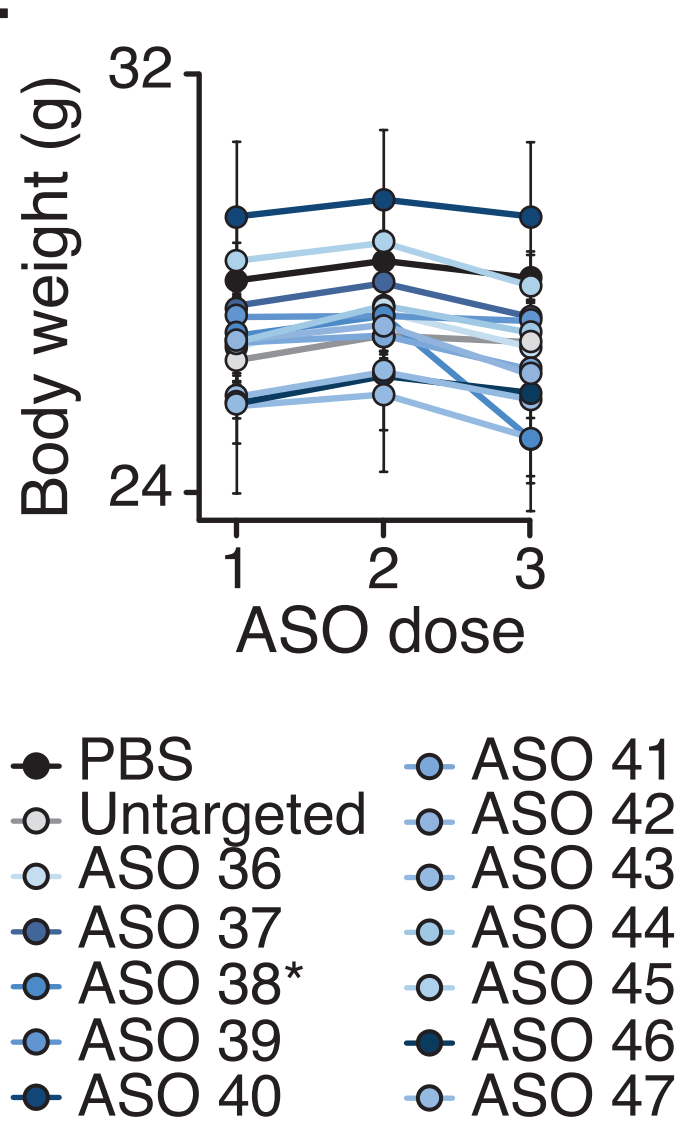

F

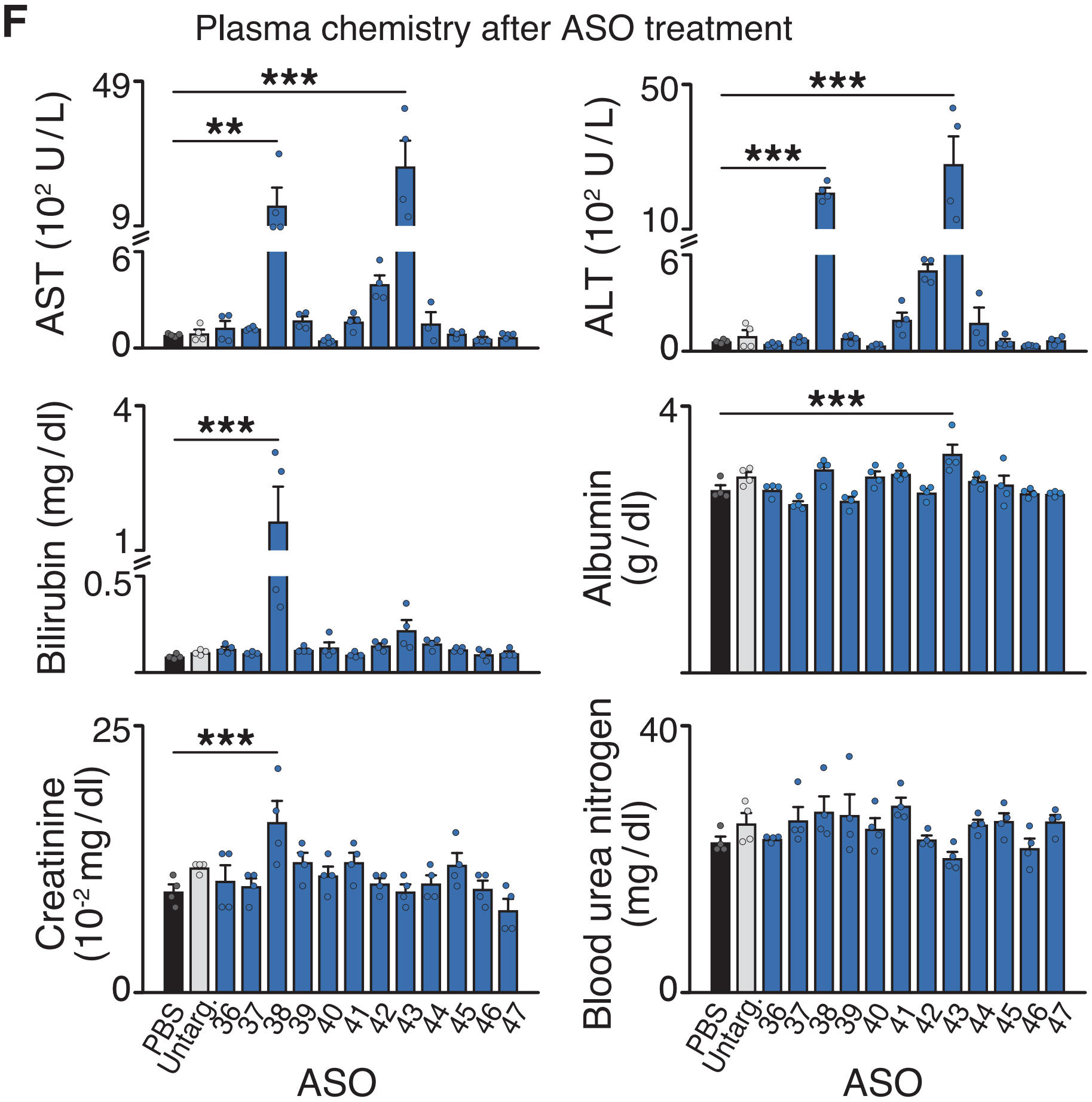

Figures

Fig. S1. Screening identifies well-tolerated adipose-targeted *Zfp423* ASOs with robust target knockdown. (A) Schematic of the *Zfp423* pre-mRNA transcript showing the binding locations of the ASOs tested. Exons are demarcated as vertical black bars. **(B)** Sequences of the untargeted ASO and *Zfp423*-targeting ASOs, along with their respective pre-mRNA target sequences. **(C)** Experimental dosing schedule for tolerability and efficacy studies. **(D)** Relative *Zfp423* mRNA expression in WAT after three doses at 50mg/kg per dose over ten day as described (n=3-4). Tissue was collected two days after the final dose. **(E)** Body weight measured prior to each dose of ASO. * Designates the treatment condition which lost weight on a chow diet. **(F)** Serum markers of liver and kidney function from plasma collected at necropsy. Data are represented as mean ± SEM. Significance was calculated by one-way ANOVA followed by Dunnett's multiple comparisons test. *$p < 0.05$, **$p<0.01$, ***$p<0.001$.

Figure S2

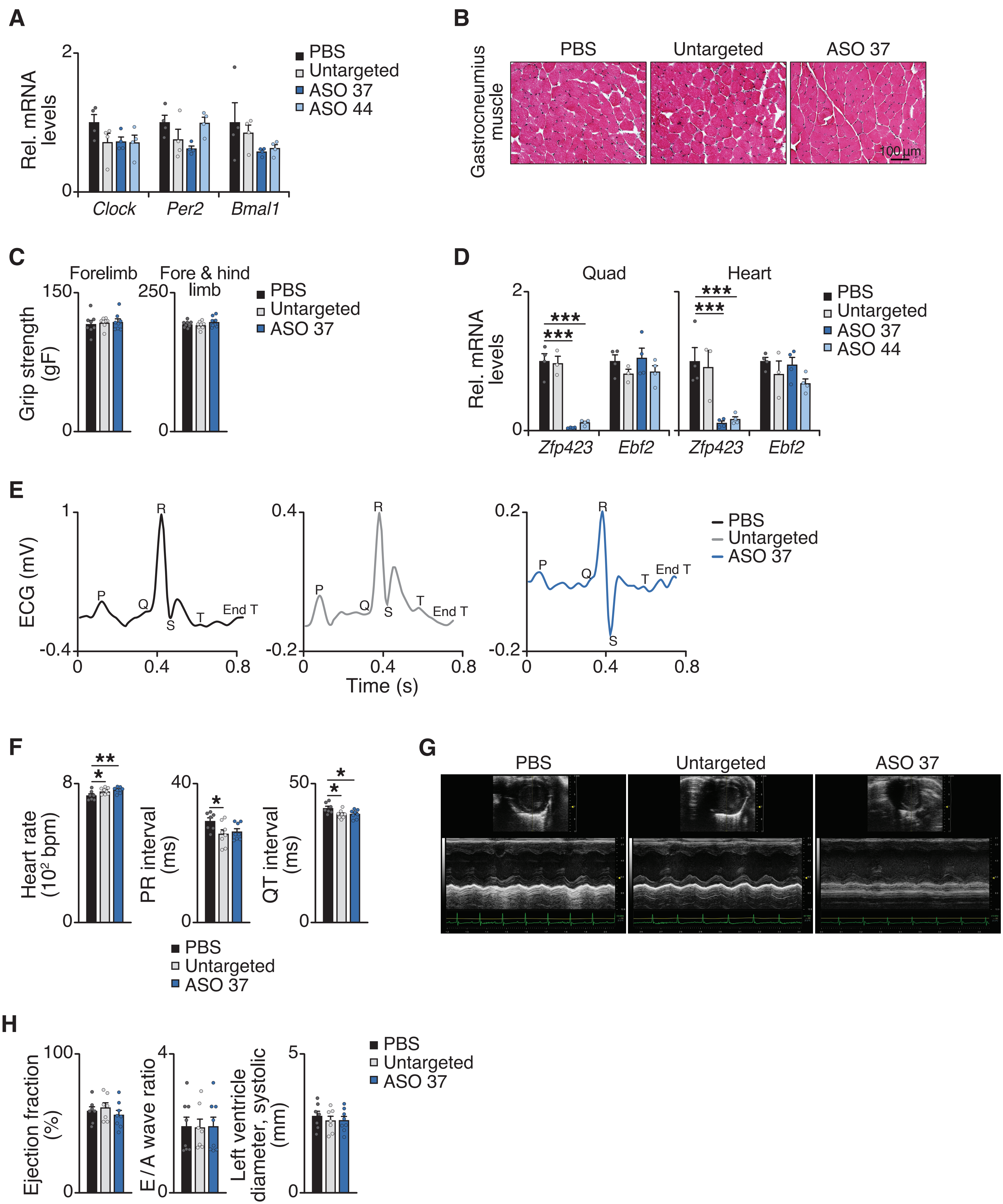

A
Rel. mRNA levels
2
0
Clock
Per2
Bmal1
PBS
Untargeted
ASO 37
ASO 44
B
Gastrocneumius muscle
PBS
Untargeted
ASO 37
100 µm
C
Forelimb
Fore & hind limb
Grip strength (gF)
150
250
0
PBS
Untargeted
ASO 37
D
Quad
Heart
Rel. mRNA levels
2
0
***
Zfp423
Ebf2
PBS
Untargeted
ASO 37
ASO 44
E
ECG (mV)
1
-0.4
0.4
-0.2
0.2
0
0.8
Time (s)
P
Q
R
S
T
End T
PBS
Untargeted
ASO 37
F
Heart rate (10² bpm)
8
PR interval (ms)
40
QT interval (ms)
50
*
**
PBS
Untargeted
ASO 37
G
PBS
Untargeted
ASO 37
H
Ejection fraction (%)
100
E/A wave ratio
4
Left ventricle diameter, systolic (mm)
5
PBS
Untargeted
ASO 37

Fig. S2. *Zfp423* ASO treatment preserves skeletal muscle and cardiac function in chow-fed mice. (A) Expression of circadian genes in iWAT from mice treated for 10 weeks on a chow-fed diet (n=4) **(B)** Representative H&E staining from gastrocnemius muscle of mice treated for 10 weeks on a chow-fed diet. **(C)** Forelimb-only and combined forelimb/hindlimb grip strength in mice treated weekly for 5 weeks measured in grams of force (gF) (n=8). **(D)** Relative mRNA levels of *Zfp423* and *Ebf2* in quad muscle and heart (n=4). **(E)** Representative electrocardiogram (ECG) traces after 4 weeks of ASO37 or control treatment. **(F)** Heart rate, PR interval, and QT interval in mice treated for 4 weeks (n=7-8). **(G)** Representative echocardiogram (echo) images and **(H)** ejection fraction, E/A wave ratio, and left ventricle diameter during systole after 4 weeks of ASO37 or control treatments (n=7-8). Data are represented as mean ± SEM. Significance was calculated one-way ANOVA followed by Tukey's multiple comparison's test where three or more conditions are compared. Where two categorical variables, a two-way ANOVA was performed followed by Tukey's multiple comparison's test. *p< 0.05, **p<0.01, ***p<0.001.

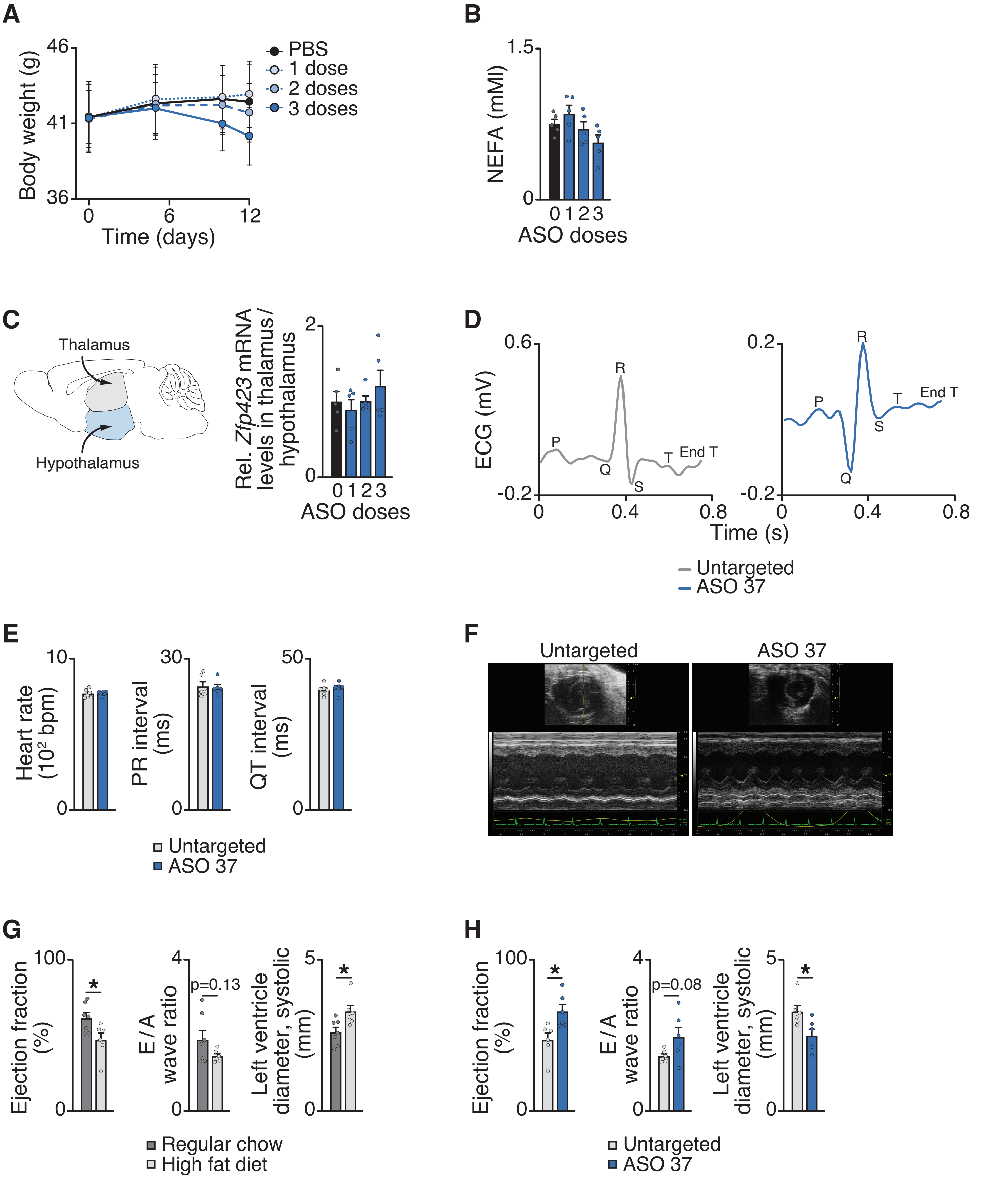
A
Body weight (g)
46
41
36
0
6
12
Time (days)
PBS
1 dose
2 doses
3 doses
B
NEFA (mMl)
1.5
0
0 1 2 3
ASO doses
C
Thalamus
Hypothalamus
Rel. Zfp423 mRNA levels in thalamus / hypothalamus
2
0
0 1 2 3
ASO doses
D
ECG (mV)
0.6
-0.2
P
Q
R
S
T
End T
0
0.4
0.8
0.2
-0.2
Time (s)
Untargeted
ASO 37
E
Heart rate (10² bpm)
10
0
PR interval (ms)
30
0
QT interval (ms)
50
0
Untargeted
ASO 37
F
Untargeted
ASO 37
G
Ejection fraction (%)
100
0
*
E/A wave ratio
4
0
p=0.13
Left ventricle diameter, systolic (mm)
5
0
*
Regular chow
High fat diet
H
Ejection fraction (%)
100
0
*
E/A wave ratio
4
0
p=0.08
Left ventricle diameter, systolic (mm)
5
0
*
Untargeted
ASO 37

Fig. S3. *Zfp423*-targeting ASOs rescue cardiac dysfunction caused by obesity and do not act centrally or alter hypothalamic/thalamic *Zfp423* expression. **(A)** Body weights plotted over the dosing period in mice treated with one, two or three doses of ASO37 (50 mg/kg per dose) or equivalent volume of PBS. Mice were fed a high fat diet for 6 weeks prior to the onset of dosing (n=5). **(B)** Serum non-esterified fatty acids (NEFAs) measured after 1, 2, or 3 doses of ASO37 (n=5). **(C)** Schematic depicting the hypothalamic/thalamic brain regions dissected for analysis, with relative *Zfp423* gene expression measured in this region after PBS or ASO37 treatment (n=5). **(D)** Representative electrocardiogram (ECG) traces after ten weeks of high fat diet (HFD) and 4 weeks of ASO37 or control treatment. **(E)** Heart rate, PR interval, and QT interval in mice treated for 4 weeks (n=6). **(F)** Representative echocardiogram (echo) images after ten weeks of HFD and 4 weeks of ASO37 or control treatment **(G)** Ejection fraction, E/A wave ratio, and left ventricle diameter in systole after 4 weeks of untargeted treatment in chow-fed and HFD-fed conditions (n=6-7) **(H)** ejection fraction, E/A wave ratio, and left ventricle diameter in systole after 4 weeks of ASO37 or control treatments (n=6). Data are represented as mean ± SEM. Significance was calculated by two-way ANOVA for panel A, one-way ANOVA for panels B and C, and Student's t-test for panel E, G, and H.

Figure S4

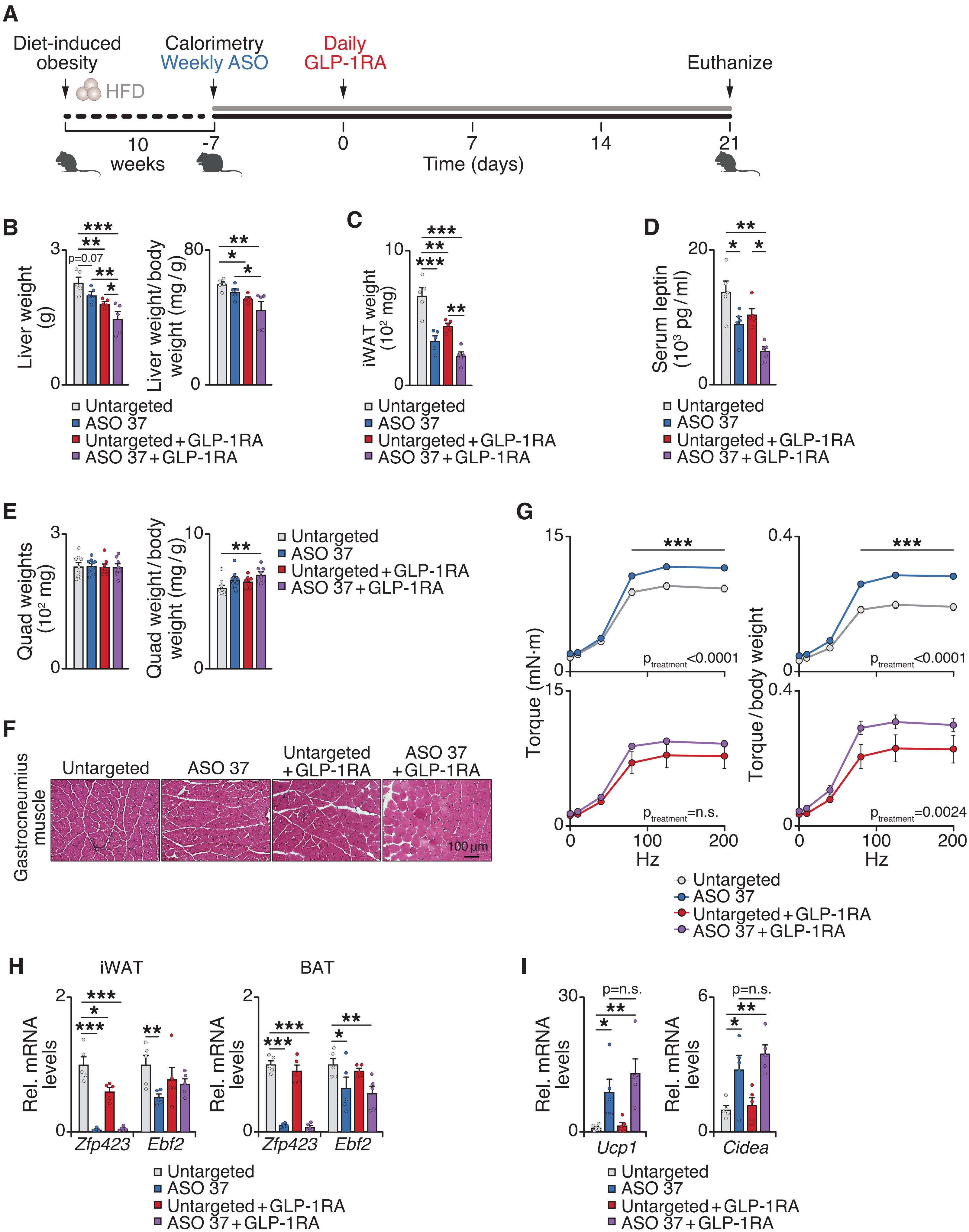
A
Diet-induced obesity
HFD
Calorimetry
Weekly ASO
Daily GLP-1RA
Euthanize
10 weeks
-7
0
7
14
21
Time (days)
B
Liver weight (g)
Liver weight/body weight (mg/g)
p=0.07
C
iWAT weight (10² mg)
D
Serum leptin (10³ pg/ml)
Untargeted
ASO 37
Untargeted + GLP-1RA
ASO 37 + GLP-1RA
E
Quad weights (10² mg)
Quad weight/body weight (mg/g)
F
Gastrocneumius muscle
Untargeted
ASO 37
Untargeted + GLP-1RA
ASO 37 + GLP-1RA
100 µm
G
Torque (mN·m)
Torque/body weight
p treatment <0.0001
p treatment =n.s.
p treatment =0.0024
Hz
H
iWAT
BAT
Rel. mRNA levels
Zfp423
Ebf2
I
p=n.s.
Ucp1
Cidea

Fig. S4. Adipose-targeted *Zfp423* ASOs improve muscle strength when used with or without a GLP-1RA. (A) Experimental timeline. **(B-C)** Liver weights, liver to body weight ratio, and iWAT weights measured at tissue collection on day 21 (n=5). **(D)** Leptin from serum collected at necropsy. **(E)** Quad weights. Left- unnormalized. Right- normalized to body weight measured at tissue collection on day 21 (n=8-9). **(F)** Representative hematoxylin and eosin (H&E) staining of gastrocnemius muscle after tissue collection on day 21. **(G)** Muscle function studies where torque (angular force) caused by a muscle contraction was measured in response to electrical stimuli provided at the given frequency (Hz) prior to tissue collection on day 21. (Top: n=5-6, bottom: n=3-4.) **(H)** Relative mRNA levels of *Zfp423* and *Ebf2* from iWAT and BAT depots collected on day 21 (n=5). **(I)** Relative mRNA levels of *Ucp1* and *Cidea* from iWAT collected on day 21 (n=5). Data are represented as mean ± SEM. Significance was calculated by two-way ANOVA followed by Tukey's multiple comparisons test where two categorical variables and one-way ANOVA followed by Tukey's multiple comparisons test where one categorical variable. *p<0.05, **p<0.01, ***p<0.001.

Figure S5

A

Pairwise comparison	Symbol
Untargeted vs ASO 37	*
Untargeted vs Untargeted+GLP-1RA	#
Untargeted vs ASO 37+GLP-1RA	+

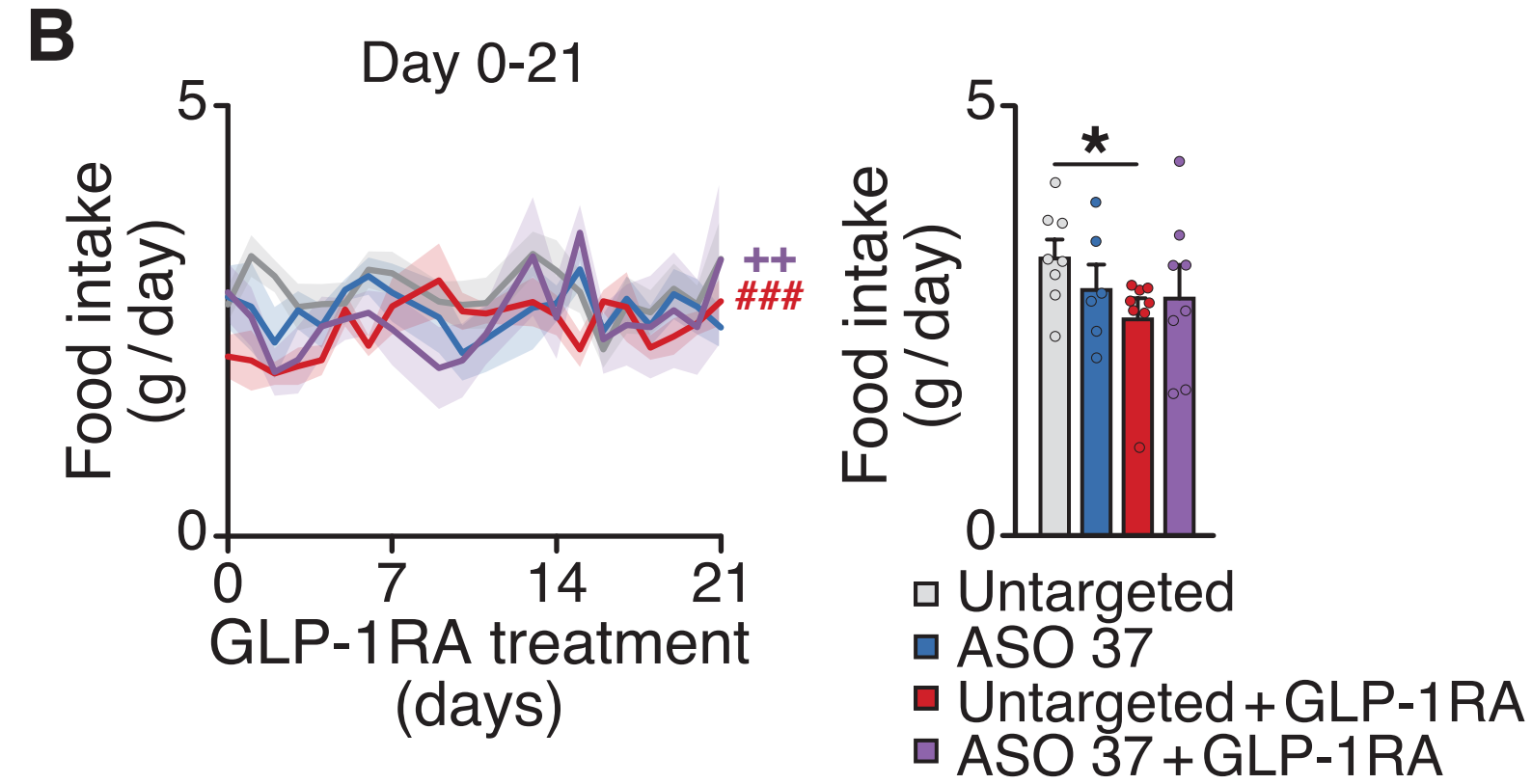

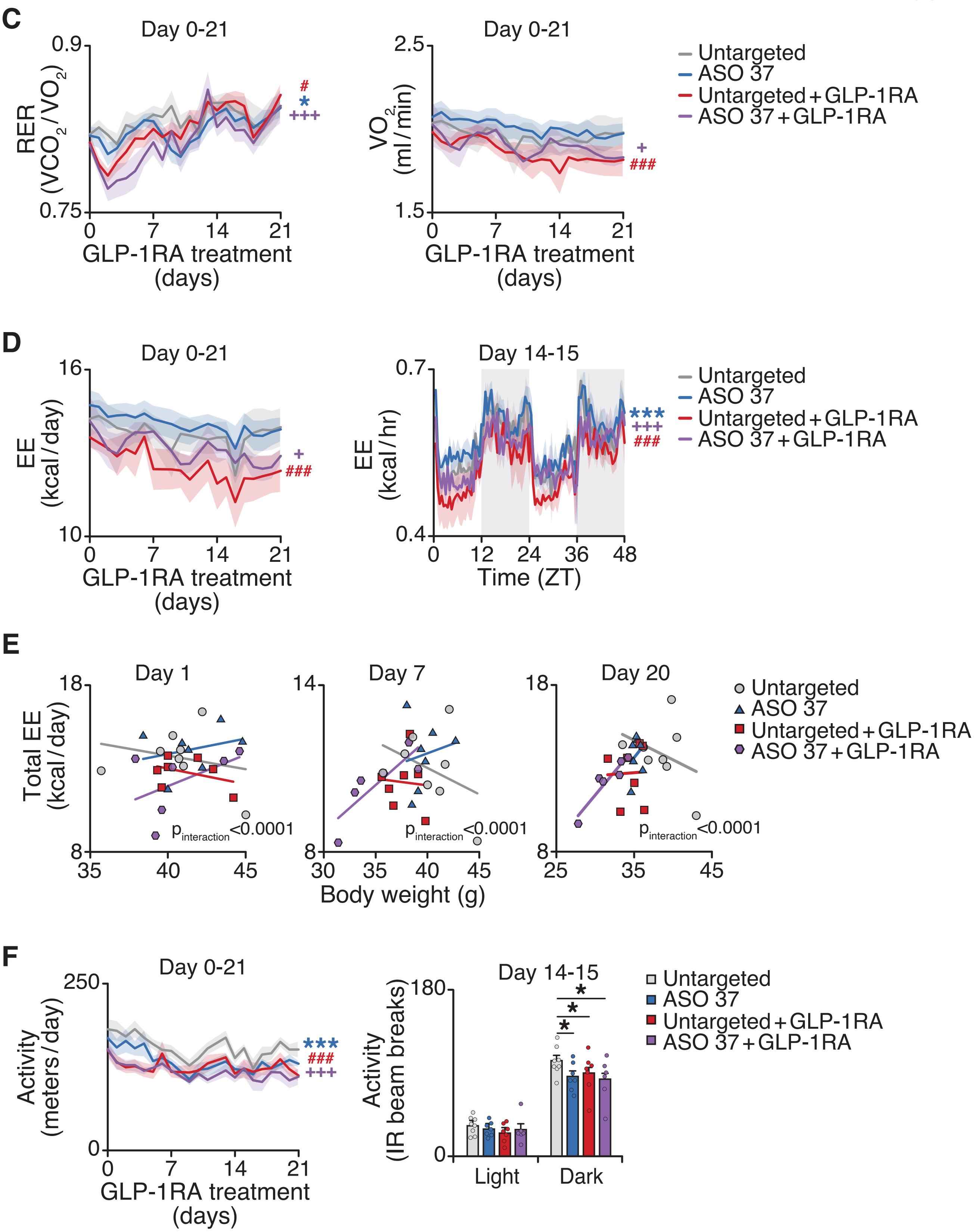

Fig. S5. Adipose-targeted *Zfp423* ASOs increase energy expenditure when used with a GLP-1RA. (A) Table explaining pairwise comparisons depicted throughout the plot. **(B)** Food intake data in Promethion Sable metabolic phenotyping chambers from diet-induced obese mice treated with untargeted ASO, ASO37, untargeted ASO + GLP-1RA, or targeted ASO37 + GLP-1RA (n=6-8). Left: Food intake data over all 21 days of GLP-1RA treatment. Right: two days of data (day 14-15) after 2 weeks of GLP-1RA treatment were plotted. **(C)** Left: Average daily respiratory exchange ratio (VCO_2/VO_2) over 21 days of GLP-1RA treatment (p-value for untargeted ASO + GLP-1RA vs targeted ASO37 + GLP-1RA <0.01, n=6-8). Right: Average daily oxygen consumption over 21 days of GLP-1RA treatment (n=6-8). **(D)** Left: total daily energy expenditure over 21 days of GLP-1RA treatment (p-value for untargeted ASO + GLP-1RA vs targeted ASO37 + GLP-1RA <0.05, n=6-8). Right: average energy expenditure after two weeks of treatment (days 14-15) (p<0.001 for all pairwise comparisons, n=6-8). **(E)** Total energy expenditure over 24 hours vs body weight after two weeks of GLP-1RA treatment analyzed with a Gaussian GLM including genotype, body mass, and their interaction (plotted); per-treatment condition slopes were estimated with emtrends and compared pairwise with Sidak-adjusted p-values. Relative to untargeted: $P_{ASO37}<0.0001$ on days 1 and 7, $P_{ASO37}<0.01$ on day 20. $P_{ASO37+GLP\text{-}1RA}<0.0001$ on days 1, 7 and 20. $P_{GLP\text{-}1RA}$=not significant on days 1 and 7, $P_{GLP\text{-}1RA}$ <0.0001 on day 21 (n=6-8). **(F)** Total daily locomotor activity over all 21 days of GLP-1RA treatment and two days of data plotted by light- and dark-period activity (day 14-15) were averaged (n=6-8). Data are represented as mean ± SEM or estimated marginal mean ± SEM for light/dark activity. Significance was calculated by two-way ANOVA followed by Tukey's multiple comparisons test where two categorical variables and one-way ANOVA followed by Dunnett's multiple comparisons test where one categorical variable. P-values for 21-day traces and two-day average energy expenditure panels were performed by two-way ANOVA for time and treatment condition with post-hoc Tukey's multiple hypothesis adjustment to test pairwise comparisons for all six treatment group comparisons. Only comparisons relative to the untargeted condition were plotted for clarity. *p< 0.05, **p<0.01, ***p<0.001, #p< 0.05, ##p<0.01, ###p<0.001, +p< 0.05, ++p<0.01, +++p<0.001.

Figure S6

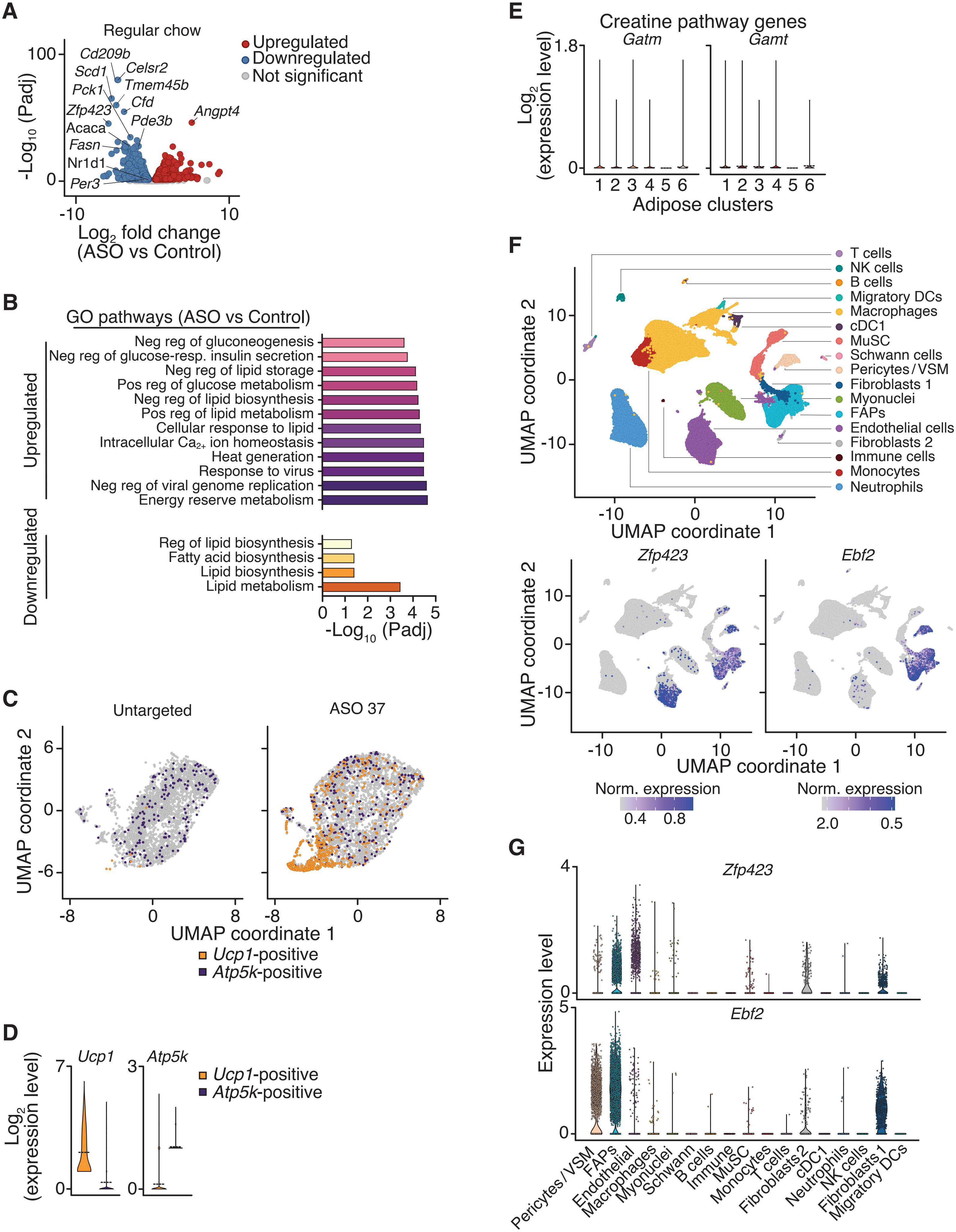
A
Regular chow
Upregulated
Downregulated
Not significant
Cd209b
Scd1
Celsr2
Tmem45b
Pck1
Cfd
Zfp423
Pde3b
Angpt4
Acaca
Fasn
Nr1d1
Per3
-Log10 (Padj)
Log2 fold change (ASO vs Control)
B
GO pathways (ASO vs Control)
Upregulated
Neg reg of gluconeogenesis
Neg reg of glucose-resp. insulin secretion
Neg reg of lipid storage
Pos reg of glucose metabolism
Neg reg of lipid biosynthesis
Pos reg of lipid metabolism
Cellular response to lipid
Intracellular Ca2+ ion homeostasis
Heat generation
Response to virus
Neg reg of viral genome replication
Energy reserve metabolism
Downregulated
Reg of lipid biosynthesis
Fatty acid biosynthesis
Lipid biosynthesis
Lipid metabolism
-Log10 (Padj)
C
Untargeted
ASO 37
UMAP coordinate 2
UMAP coordinate 1
Ucp1-positive
Atp5k-positive
D
Ucp1
Atp5k
Log2 (expression level)
Ucp1-positive
Atp5k-positive
E
Creatine pathway genes
Gatm
Gamt
Log2 (expression level)
Adipose clusters
F
T cells
NK cells
B cells
Migratory DCs
Macrophages
cDC1
MuSC
Schwann cells
Pericytes / VSM
Fibroblasts 1
Myonuclei
FAPs
Endothelial cells
Fibroblasts 2
Immune cells
Monocytes
Neutrophils
UMAP coordinate 2
UMAP coordinate 1
Zfp423
Ebf2
Norm. expression
Norm. expression
G
Zfp423
Ebf2
Expression level
Pericytes / VSM
FAPs
Endothelial
Macrophages
Myonuclei
Schwann
B cells
Immune
MuSC
Monocytes
T cells
Fibroblasts 2
cDC1
Neutrophils
NK cells
Fibroblasts 1
Migratory DCs

Fig S6. Distinct adipocyte populations express *Ucp1* and creatine biosynthetic programs. (A) Volcano plot and **(B)** GO pathway analysis of iWAT RNA-seq in chow-fed mice treated for 10 weeks with weekly 50 mg/kg doses of ASO37 or untargeted control (n=4). **(C)** UMAP of adipocyte nuclei highlighting Ucp1-positive and Atp5k-positive subpopulations, segregated by treatment condition. **(D)** Violin plots of *Ucp1* expression (left) and *Atp5k* expression (right) within Ucp1-positive and Atp5k-positive adipocyte populations. p<0.01 by Fisher's exact test. **(E)** Violin plots showing G*atm* and G*amt* expression across adipocyte clusters with minimal expression in cluster 5. **(F)** UMAP of single cell RNA-seq dataset depicting muscle cell types and expression of *Zfp423* and *Ebf2* in muscle. **(G)** Violin plots quantifying *Zfp423* and *Ebf2* expression within muscle cell populations. Median is demarcated on violin plots with a dotted line for adipose plots. Cells are depicted as dots for muscle violin plots. Adjusted p-value is used for volcano plots.

Tables

Table S1. Table containing full ASO product numbers, sequences and simple chemistry

Ionis ASO	ASO Equivalent Sequence	Pre-mRNA Targeting Sequence	Simple Chemistry
Untargeted 1694086	5'-CGCCGATAAGGTACAC-3	N/A	palmamnC6o-oKKK-10-KKK
1892536	5'-AATGTTTAACGAGTCA-3'	5'-UGACUCGUUAAACAUU-3'	palmamnC6o-oKKK-10-KKK
1892537	**5'-TTTTTATTACAGCGGA-3'**	**5'-UCCGCUGUAAUAAAAA-3'**	palmamnC6o-oKKK-10-KKK
1892538	5'-AGAGATAATTCAGCGG-3'	5'-CCGCTUGAAUUAUCUCU-3'	palmamnC6o-oKKK-10-KKK
1892539	5'-GACATTAAGGTACTGC-3'	5'-GCAGUACCUUAAUGUC-3'	palmamnC6o-oKKK-10-KKK
1892540	5'-ATTTTAGCGAGGTTCG-3'	5'-CGAACCUCGCUAAAAU-3'	palmamnC6o-oKKK-10-KKK
1892541	5'-GGTATTAGAACAATTC-3'	5'-GAAUUGUUCUAAUACC-3	palmamnC6o-oKKK-10-KKK
1892542	5'-TCTGATAAGGCTTGGC-3'	5'-GCCAAGCCUUAUCAGA-3'	palmamnC6o-oKKK-10-KKK
1892543	5'-AAGGATACAAGACTGG-3'	5'-CCAGUCUUGUAUCCUU-3'	palmamnC6o-oKKK-10-KKK
1892544	**5'-ACCATAAGATTCACCA-3'**	**5'-UGGUGAAUCUUAUGGU-3'**	palmamnC6o-oKKK-10-KKK
1892545	5'-ACTGATTTAACACACT-3'	5'-AGUGUGUUAAAUCAGU-3'	palmamnC6o-oKKK-10-KKK
1892546	5'-CATAAATCTGATGCGG-3'	5'-CCGCAUCAGAUUUAUG-3'	palmamnC6o-oKKK-10-KKK
1892547	5'-CCAGAATTGATACCCA-3'	5'-UGGGUAUCAAUUCUGG-3'	palmamnC6o-oKKK-10-KKK

Table S2. Table containing qPCR primer sequences used

Gene	Forward	Reverse
Zfp423	CAGGCCCACAAGAAGAACAAG	GTATCCTCGCAGTAGTCGCACA
Ebf2	CCAGCTCTGAAAGACAAGTCG	GAGGTTGCTTTTCAAAATGGGC
Adipoq	AGATGGCACTCCTGGAGAGAA	TTCTCCAGGCTCTCCTTTCCT
Cidea	TCCTATGCTGCACAGATGACG	TGCTCTTCTGTATCGCCCAGT
Prdm16	ACACGCAGTTCTCCAACCTGT	CCCACAGTCCTTGCACTTGAT
Dio2	CATTGATGAGGCTCACCCTTC	GGTTCCGGTGCTTCTTAACCT
Ucp1	TCTCAGCCGGCTTAATGACTG	GGCTTGCATTCTGACCTTCAC
Clock	ACCACAGCAACAGCAACAAC	GGCTGCTGAACTGAAGGAAG
Per2	TGTGCGATGATGATTCGTGA	GGTGAAGGTACGTTTGGTTTGC
Bmal1	AGGCCCACAGTCAGATTGAA	TGGTACCAAAGAAGCCAATTCAT

Movie S1. Echocardiogram videos from mice treated with ASO for four weeks (A) PBS-treated **(B)** Untargeted ASO-treated **(C)** ASO37-treated

Movie S2. Echocardiogram videos from diet-induced obese mice treated with ASO for four weeks (A) Untargeted ASO-treated **(B)** ASO37-treated

SI References

References:

1. Barefield, D.Y., et al., *Experimental Modeling Supports a Role for MyBP-HL as a Novel Myofilament Component in Arrhythmia and Dilated Cardiomyopathy.* Circulation, 2017. **136**(16): p. 1477-1491.
2. Zelikovich, A.S., et al., *Moderate exercise improves function and increases adiponectin in the mdx mouse model of muscular dystrophy.* Sci Rep, 2019. **9**(1): p. 5770.
3. Ramachandran, K., et al., *Transcriptional programming of translation by BCL6 controls skeletal muscle proteostasis.* Nat Metab, 2024. **6**(2): p. 304-322.
4. Keeble, A.R., et al., *CSF1-R inhibition attenuates posttraumatic osteoarthritis and quadriceps atrophy following ligament injury.* J Physiol, 2025. **603**(23): p. 7549-7566.
5. Langer, H.T., et al., *Weight loss with GLP-1 medicines does not result in a disproportionate loss of muscle mass or function in obese mice and humans.* Cell Rep Med, 2026. **7**(3): p. 102665.
